# Photodynamic therapy for cancer: mechanisms, photosensitizers, nanocarriers, and clinical studies

**DOI:** 10.1002/mco2.603

**Published:** 2024-06-22

**Authors:** Wanchen Zhao, Liqing Wang, Meihong Zhang, Zhiqi Liu, Chuanbin Wu, Xin Pan, Zhengwei Huang, Chao Lu, Guilan Quan

**Affiliations:** ^1^ State Key Laboratory of Bioactive Molecules and Druggability Assessment Jinan University Guangzhou China; ^2^ College of Pharmacy Jinan University Guangzhou China; ^3^ School of Pharmaceutical Sciences Sun Yat‐sen University Guangzhou China

**Keywords:** clinical studies, molecular mechanisms, nanocarriers, PDT, photosensitizers

## Abstract

Photodynamic therapy (PDT) is a temporally and spatially precisely controllable, noninvasive, and potentially highly efficient method of phototherapy. The three components of PDT primarily include photosensitizers, oxygen, and light. PDT employs specific wavelengths of light to active photosensitizers at the tumor site, generating reactive oxygen species that are fatal to tumor cells. Nevertheless, traditional photosensitizers have disadvantages such as poor water solubility, severe oxygen‐dependency, and low targetability, and the light is difficult to penetrate the deep tumor tissue, which remains the toughest task in the application of PDT in the clinic. Here, we systematically summarize the development and the molecular mechanisms of photosensitizers, and the challenges of PDT in tumor management, highlighting the advantages of nanocarriers‐based PDT against cancer. The development of third generation photosensitizers has opened up new horizons in PDT, and the cooperation between nanocarriers and PDT has attained satisfactory achievements. Finally, the clinical studies of PDT are discussed. Overall, we present an overview and our perspective of PDT in the field of tumor management, and we believe this work will provide a new insight into tumor‐based PDT.

## Graphical abstract

We highlight the development and molecular mechanisms underlying the activation of photosensitizers, the challenges of PDT in tumor management, and the advantages of nanocarriers‐based PDT against cancer.

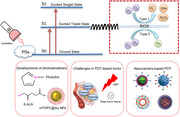

## 1 INTRODUCTION

The World Health Organization reports that cancer is responsible for approximately 10 million deaths each year, making it the second leading cause of mortality worldwide.^1^, ^2^ Currently, the main clinical treatments for cancer are cytoreductive or suppressive therapies based on surgical resection, chemotherapy, radiotherapy, or a combination of these therapies. Due to the risk of recurrence, tumor invasiveness, and side effects caused by the lack of tumor‐specific targeting, the efficacy of these treatments remains inadequate.^3^, ^4^ As a result, the emergence of new therapies has been driven by clinical need.^5^, ^6^ Several laser‐based therapies, including photodynamic therapy (PDT) and photothermal therapy (PTT), have also been developed based on the concept of precision tumors treatment.

PDT is a promising noninvasive therapy that has demonstrated distinct advantages in recent years for combating bacteria,^7^, ^8^ fungi,^9^, ^10^ viruses,^11^, ^12^ and particularly cancers.^13^, ^14^, ^15^ Compared with traditional therapeutics (e.g., surgery), PDT has the following advantages: (i) minimally invasive nature, (ii) few side effects, (iii) high temporal and spatial controllability, and (iv) no obvious drug resistance.^16^ In practice, photosensitizers (PSs) enriched near tumor cells can be activated by irradiation with specific wavelengths of light, resulting in the generation of reactive oxygen (O_2_) species (ROS). These ROS include singlet oxygen (^1^O_2_), superoxide anion radicals (O_2_^•−^), hydrogen peroxide (H_2_O_2_), and hydroxyl radicals (•OH), which kill tumors mainly by inducing apoptosis or necrosis.^17^, ^18^

PSs, light, and O_2_ are indispensable in PDT. Therefore, they are termed the three elements of PDT, and PSs are considered as key factors affecting the efficacy of PDT. It is generally believed that ideal PSs should have the advantages of a single component, high stability, low dark toxicity, high solubility, high intersystem channeling efficiency, and selective retention in the target tissue.^16^, ^19^ The excitation wavelengths of mainstream PSs are all distributed in the range of 600−900 nm, which is known as the biological window and is the least absorbed by water and biomolecules; therefore, this type of light can penetrate and reach deep tissues.^20^ In addition, the ground‐state PS absorbs photon energy, transitions to the excited triplet state, and then produces cytotoxic ROS, in which molecular O_2_ is an essential reaction substrate for PDT, and sufficient O_2_ is very important for the efficiency of PDT.^21^ Unfortunately, in solid tumors, the tumor microenvironment (TME) is characterized by overall hypoxia due to the rapid growth of tumor tissue, high volume expansion, and an incomplete vascular system inside the tissue.^22^ Currently, most PSs reported in the literature are type II PSs that produce type II ROS (i.e., ^1^O_2_) through energy transfer. The effect of this PS is highly dependent on the O_2_ concentration in the environment; therefore, a lack of O_2_ in the TME seriously affects the therapeutic effect of PDT. On the contrary, type I PSs produce ROS (including O_2_^•−^, H_2_O_2_, and •OH) through electron or proton transfer, which is less dependent on O_2_ and can effectively kill tumor cells, even in the absence of O_2_. This contributes to the recognized advantages of PDT anticancer treatment.^23^

In addition to hypoxia, accumulating shreds of evidence have revealed that the TME is an incredibly cunning system significantly characterized by immunosuppression, low extracellular pH (pHe), high H_2_O_2_ concentration, overexpression of enzymes, and so on, which together determine the uncontrolled physiological characteristics of the TME and confer tumors heterogeneity, ultimately contribute to drug resistance of tumor cells.^24^ Hypoxia in TME dramatically impedes the therapeutic effect of PDT, while O_2_ depletion during PDT treatment exacerbates tumor hypoxia, and synergistically performs severe side effects after PDT treatment.^25^ Additionally, the abnormality of the TME makes it difficult to deliver PSs effectively.

To overcome the limitations of PDT, significant efforts are being made to explore novel therapeutic strategies that can facilitate the application of PDT in cancer treatment. This review provides a comprehensive overview of the evolution of PSs, the molecular mechanisms underlying the activation of PSs, and the current challenges faced by PDT in tumor management. Notably, this review emphasizes the pivotal role played by recent nanotechnological breakthroughs and nanocarriers in overcoming conventional PSs’ limitations. Specifically, it highlights the advantages offered by various nanocarriers in improving the water solubility of PSs, overcoming wavelength limitations for deep tissue PDT, enhancing tumor‐specific delivery of PSs, increasing PDT efficacy in hypoxic environments, and facilitating combination therapy strategies. Advances in the clinical applications of PDT are also specifically summarized. We anticipate that this work will serve as a tutorial guide and a valuable reference for future advancements in PDT (particularly, nanocarriers‐involved) for tumor management.

## 2 THE BASICS AND MECHANISMS OF PDT FOR CANCER

The antitumor efficacy of PDT relies on the generation of ROS through the interaction of PSs, light, and O_2_. PSs can engage in either type I or type II reactions, or a combination of both, to destroy cancer cells. The ratio of these two types of reactions depends on the specific PSs utilized. If a PS primarily generates free radicals through type I reactions, it may be more effective in cellular environments rich in biomolecules; whereas if it primarily generates ^1^O_2_ through type II reactions, it may be more effective in environments with higher O_2_ concentrations.^26^

The biological mechanisms of PDT in cancer therapy are typically reflected in three aspects: (1) direct killing: ROS generated during PDT exert a toxic effect on tumor cells, inducing oxidative stress, damage, apoptosis, or necrosis, thus achieving the purpose of cancer treatment^27^; (2) vascular closure: PDT acts on the blood vessels surrounding the tumor tissues, resulting in tumor cell death due to ischemia and O_2_ deprivation^28^; (3) inducing immune effects: PDT is also capable of inducing immunogenic cell death (ICD), promoting the release of tumor‐associated antigens, activating and enhancing the body's immune response. Ultimately, this exerts a therapeutic effect on distant tumor cells such as residual or metastatic ones.^29^, ^30^

## 3 THE DEVELOPMENT COURSE OF PSs

**FIGURE 1:**
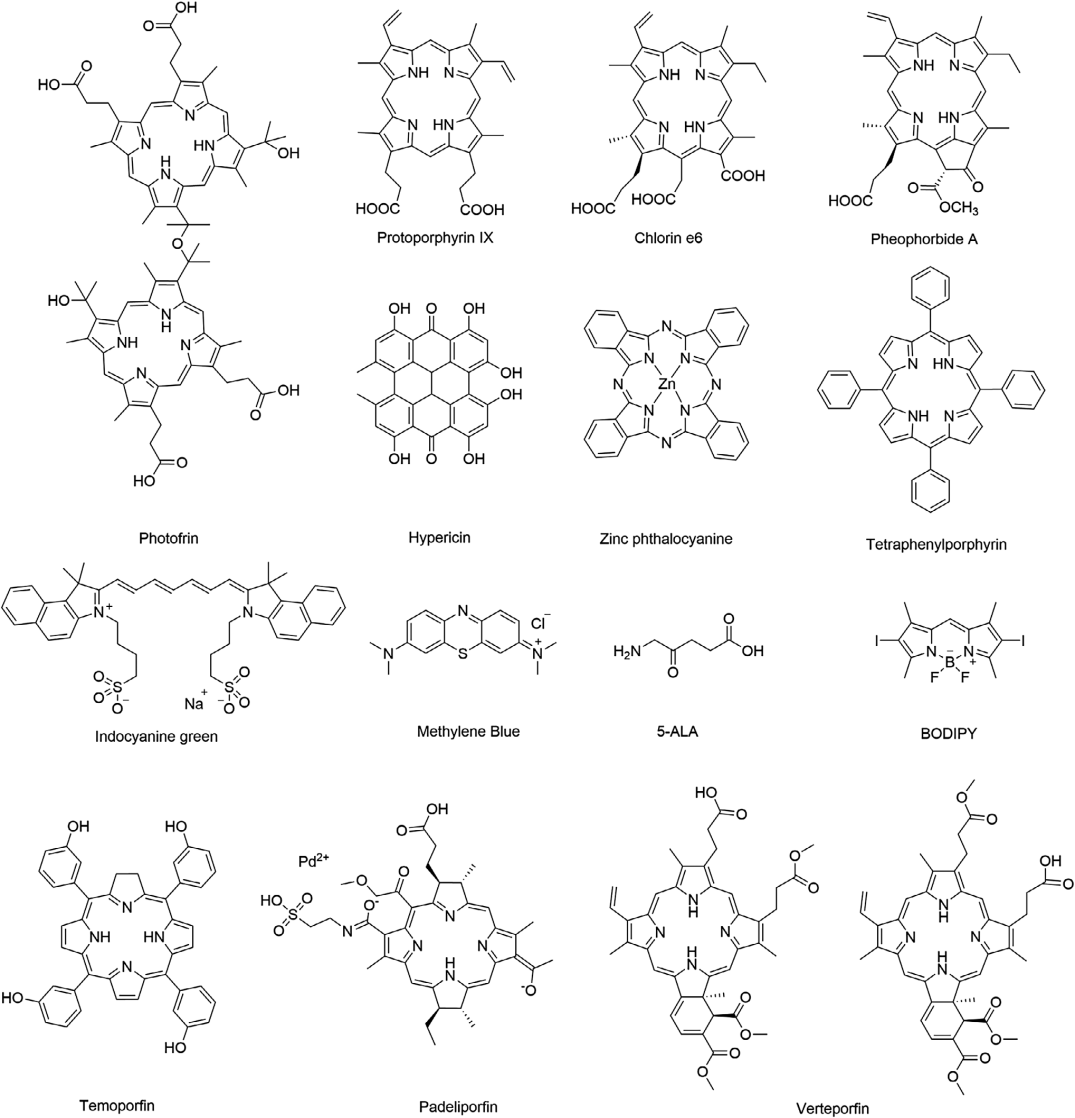
Molecular structures of some classical PSs. 5‐ALA, 5‐aminolevulinic acid; BODIPY, boron dipyrromethene. This figure is drawn by authors using ChemDraw.

The PSs that have been developed to date can be divided into three generations (Tables 1 and 2, Figure 1). First‐generation PSs primarily include hematoporphyrin derivative, dihematoporphyrin ether, and porfimer sodium (Photofrin^®^). In 1950, Schwartz discovered that hematoporphyrin can be selectively enriched in tumor tissues.^31^ This also promoted the development of first‐generation PSs based on hematoporphyrin.^32^, ^33^ By the 1970s, Photofrin^®^, isolated from a hematoporphyrin mixture, was approved by the United States Food and Drug Administration (US FDA) for cancer treatment.^34^ Although first‐generation PSs have made positive progress in the treatment of body surface tumors, they have many recognized shortcomings, including high skin phototoxicity, low ^1^O_2_ production, low extinction coefficient in the near‐infrared (NIR) region, and obvious individual differences in clinical treatment effects.^35^, ^36^

**TABLE 1:** Common first‐generation and second‐generation PSs.

PS	Generation	Excitation wavelength (nm)	Quantum yield of ^1^O_2_ (*Φ*_Δ_)	Molar extinction coefficient (M^−^1 cm^−1^)	References
Photofrin	First	630.0	0.110	3.00 × 10^3^	37
HiPorfin	First	630.0	0.124	1.26 × 10^3^	37, 38
mTHPC	Second	650.0	0.300	3.00 × 10^4^	39
Verteporfin	Second	689.0	0.78	4.00 × 10^4^	40
Padeliporfin	Second	755.0	0.230	1.09 × 10^5^	41
Magnesium phthalocyanine	Second	682.0	0.290	1.35 × 10^5^	42
Chlorin e6	Second	654.0	0.650	3.80 × 10^4^	43
Mono‐l‐asparty Ce6	Second	654.0	0.770	4.00 × 10^4^	44
Zinc phthalocyanine	Second	670.0	0.670	1.40 × 10^5^	45
Aluminum tetracarboxy‐phthalocyanines	Second	605.0	0.010	1.00 × 10^4^	46
3‐(1′‐Hexyloxyethyl)−3‐devinyl pyropeophorbide‐a	Second	661.0	NA	4.70 × 10^4^	47
Pheophorbide A	Second	667.0	NA	4.45 × 10^4^	48
P‐bromo‐phenylhydrazone‐methyl pyropheophorbide‐a	Second	683.0	0.292	7.03 × 10^4^	49
Pyropheophorbide methyl ester	Second	666.8	0.190	4.96 × 10^6^	50
Methylene blue	Second	660.0	0.520	6.79 × 10^4^	51
Rose bengal	Second	548.0	0.750	9.04 × 10^4^	52
Hypericin	Second	595.0	0.730	2.28 × 10^4^	53

Abbreviations: Ce6, chlorin e6; mTHPC, 5,10,15,20‐tetrakis(m‐hydroxyphenyl)chlorin; NA, not reported in the literature; PS, photosensitizer; ^1^O_2_, singlet oxygen.

**TABLE 2:** Representative examples of third‐generation PSs.

Carrier	PS	Size (nm)	Excitation wavelength (nm)	Target cell line	Indication	References
Liposomes	Ferrous chlorophyllin	25.4–80.3	652.0	B_16_·F_10_ melanoma/MLF1	Cutaneous melanoma	54
Liposomes	Cyanine	115.0	660.0	MCF‐7	Breast cancer	55
Liposomes/cell membrane	Verteporfin	120.0	690.0	4T1	Breast cancer	56
Liposomes	Ferrous chlorophyllin	59.0–149.0	652.0	B_16_ F_10_	Melanoma	54
Phospholipid	Porphyrin	100.0	675.0	MIA Paca‐2	Pancreatic cancer	57
Oxidized bletilla striata polysaccharide microcapsules	Ce6	∼200.0	808.0	4T1	Breast cancer	58
Folic acid	Ce6	120.8	660.0	MDA‐MB‐231	Breast cancer	59
Amphipathic peptide	2‐(1'‐Hexyloxyethyl)−2‐devinylpyropheophorbide‐a (HPPH)	110.0	665.0	HeLa	Cervical cancer	60
Polymeric micelles	ICG	NA	810.0	HuH‐7	Hepatocellular carcinoma	61
dendrimers	Phthalocyanine	137.2 ± 0.6	660.0	4T1	Breast cancer	62
Micelles	BODIPY‐Ir	119.2 ± 10.8	660.0	4T1	Breast cancer	63
duplex and quadruplex DNA	[{Ru(TAP)_2_}_2_(tpphz)]^4+^	NA	900.0	C8161	Human melanoma	64
Zeolitic imidazolate framework‐8	BODIPY	250.0	556.0	4T1	Breast cancer	65
Au nanoparticles	mTHPC	12 ± 0.5	650.0	SH‐SY5Y	Neuroblastoma of the brain	66
MSN	Carbon dots	40.0	640.0	4T1	Breast cancer	67
GQD	Riboflavin	3.4‐6.6	365.0	KB	Oral epidermal cancer	68
Carbon nanotubes	Ce6	191 ± 4.6	660.0	Caco‐2	Colon cancer	69
GO	Sinoporphyrin sodium	50.0	485.0	U87MG	Human glioma	70
Au, C_60_	C_60_	137.5	532.0/808.0	MCF‐7	Breast cancer	71
Fe_3_O_4_/SiO_2_	Curcumin	20.0‐60.0	450.0	4T1	Breast cancer	72
UCNPs	Ce6	28.7	980.0	4T1	Breast cancer	73
TiO_2,_ PDA	Ce6	165.4	671.0	4T1	Breast cancer	74

Abbreviations: BODIPY‐Ir, boron dipyrrole methylene‐Ir; Ce6, chlorin e6; GO, graphene oxide; GQD, graphene quantum dot; ICG, indocyanine green; MSN, mesoporous silica nanoparticles; mTHPC, 5,10,15,20‐tetrakis(m‐hydroxyphenyl)chlorin; NA, not reported in the literature; PDA, polydopamine; PS, photosensitizer; TAP, 4,5,8‐tetraazaphenanthrene; tpphz, tetrapyrido‐[3,2‐a:2′,3′‐c:3′′,2′′‐h:2′′′,3′′′‐j]phenazine; UCNPs, up‐conversion nanoparticles.

Considering the shortcomings of first‐generation PSs, monomeric compounds with high absorption and ^1^O_2_ yields in the NIR region, which were developed in the late 1980s, are collectively referred to as second‐generation PSs. Second‐generation PSs are predominantly porphyrin derivatives (e.g., dihydroporphyrin derivatives),^75^ phenothiazines (such as methylene blue and toluidine blue O),^21^, ^76^, ^77^ phthalocyanines (e.g., zinc phthalocyanine),^78^, ^79^ and other types of PSs (e.g., indocyanine green; ICG).^80^, ^81^ Currently, some second‐generation PSs (e.g., ICG, 5,10,15,20‐tetrakis(m‐hydroxyphenyl)chlorin (mTHPC) and 5‐aminolevulinic acid (5‐ALA)) are used in the clinic because of their excellent properties, but their performance in terms of local tissue penetration and tumor targeting is still unsatisfactory.

At the beginning of the 21st century, the nanotechnology revolution had a major impact on the PDT field.^82^, ^83^, ^84^ Based on second‐generation PSs, scientists have introduced carriers with biological properties or molecular recognition functions into their structures to obtain third‐generation PSs with high selectivity for focal tissues. These vectors mainly include specific targeting small molecules,^59^ polymers,^85^ polysaccharides,^58^ peptides,^60^ and other compounds, including liposomes,^86^ gold nanoparticles,^87^ mesoporous silica nanoparticles, carbon nanotubes,^69^ up‐conversion nanoparticles (UCNPs),^73^ and other nanocarriers. In addition, some nanoparticles and organic dyes can also act as PSs by absorbing light and generating ROS, such as fullerene (C_60_),^88^ TiO_2,_^74^ boron dipyrromethene (BODIPY),^65^ and PSs with aggregation‐induced emission (AIE) properties.^89^, ^90^, ^91^, ^92^ Compared with second‐generation PSs, third‐generation PSs have a larger molar extinction coefficient absorption, higher fluorescence quantum efficiency, good photostability, and better selectivity. However, research on third‐generation PSs is still in the animal experimentation stage.

## 4 MOLECULAR MECHANISMS OF VARIOUS PS ACTIVATION

### 4.1 Types of PSs

Due to the pivotal role of PSs in ROS generation, the development of effective PSs is crucial for the practical application of PDT.^93^ To date, numerous types of PSs have been developed through the relentless efforts of researchers.^94^, ^95^ These PSs can be categorized into two distinct groups based on their composition: inorganic and organic ones.^96^ Inorganic PSs mainly include noble metal nanoparticles and metal oxides, noble metal complexes, carbon nanomaterials, and graphene‐like materials.^97^, ^98^, ^99^ These PSs primarily produce type I ROS, which offer advantages such as stable performance and anti‐photo‐bleaching.^100^ Organic PSs primarily encompass tetrapyrrole compounds, other organic dyes, conjugated polymers (CPs), AIE molecules, and natural photoactive compounds derived from plants.^101^, ^102^, ^103^, ^104^ These PSs possess advantages and characteristics such as excellent stability, favorable biocompatibility, facile degradation, easy structural modification, versatile functionality, and so on, which have propelled extensive research efforts and led to the commercialization of clinical drugs.^105^ In this section, we provide an overview of the molecular mechanisms underlying various PS activation for ROS generation and outline the role played by PSs in PDT.

### 4.2 Molecular mechanisms of inorganic PSs activation

#### 4.2.1 Noble metal nanoparticles and metal oxides

**FIGURE 2:**
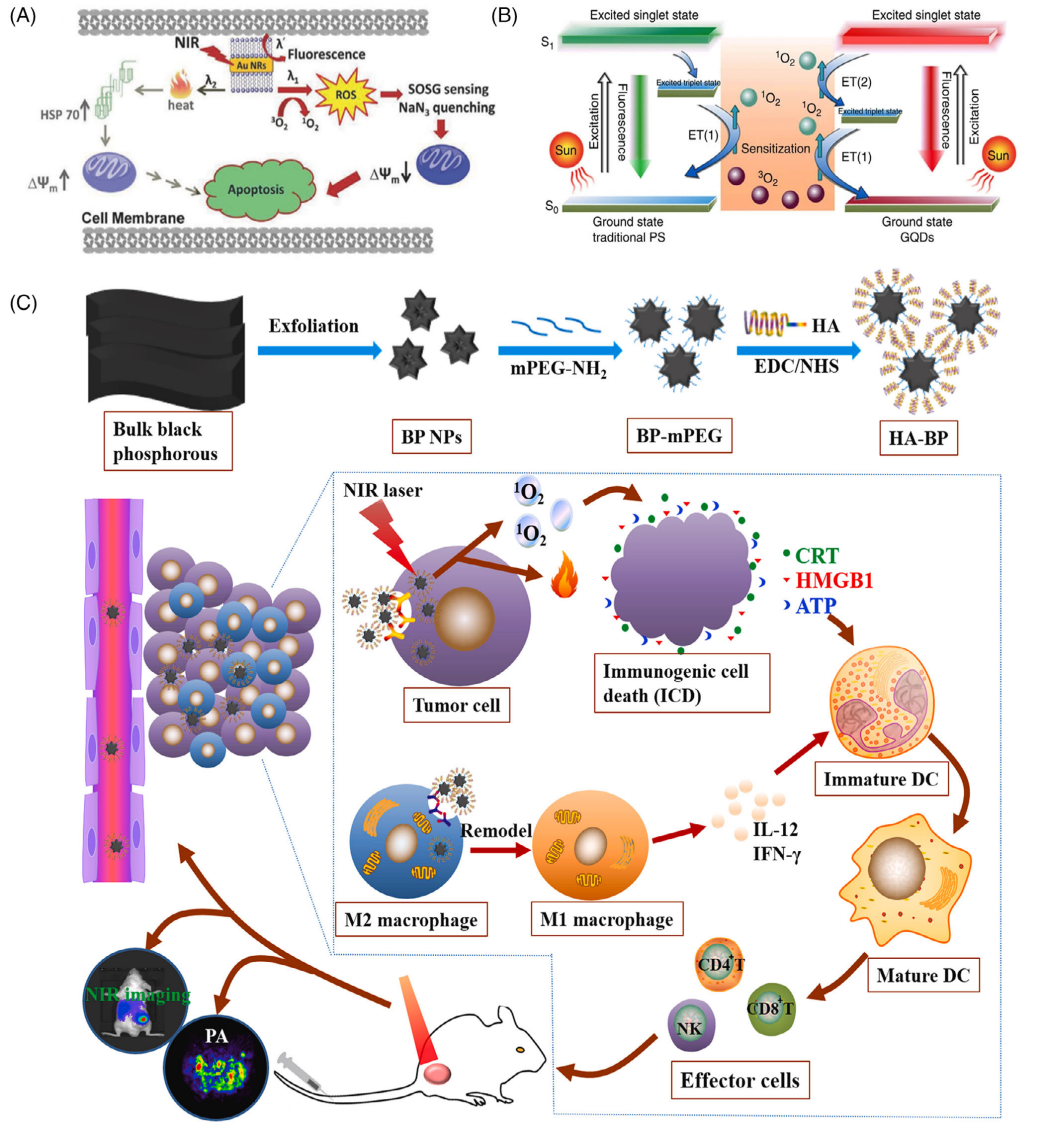
Schematic representation of PDT based on inorganic materials. (A) Series of cellular events are involved in the PDT and PTT‐induced cellular deaths upon photo‐excitation of cells internalized with Au NRs.^106^ Copyright 2013, Wiley‐VCH Verlag GmbH & Co. KGaA, Weinheim; (B) schematic illustration of the ^1^O_2_ generation mechanisms by conventional PDT agents (left) and GQDs (right).^125^ Copyright 2014, Springer Nature; (C) synthetic scheme of HA‐BP nanoparticles and the in vivo mechanism of action of HA‐BP nanoparticles.^132^ Copyright 2020, Elsevier BV. △Ψm, mitochondrial membrane potential; ^1^O_2_, singlet oxygen; ^3^O_2_, diradical; ATP, adenosine triphosphate; BP NPs, black phosphorus nanoparticles; CD4^+^/CD8^+^ T cells, specific effector T cells; CRT, calreticulin; DCs, dendritic cells; EDC·HCl, carbodiimide hydrochloride; ET, energy transfer; HA, hyaluronic acid; HMGB1, high‐mobility group protein B1; HSP 70, heat shock protein; IFN‐γ, interferon γ; IL‐12, interleukin 12; M1, activated macrophages; M2, alternatively activated macrophages; mPEG‐NH_2_, methoxypolyethylene glycol amine; NHS, N‐hydroxysuccinimide; NIR, near‐infrared region; NK, natural killer cell; PS, photosensitizer; ROS, reactive oxygen species; SOSG, oxygen sensor green.

Noble metal (mainly Au and Ag) nanoparticles exhibit localized surface plasmon resonance properties. These enable rapid and efficient energy transfer from a metal surface to molecular O_2_ and the formation of ^1^O_2_, which is a typical type‐II PDT process.^106^, ^107^, ^108^ The tunable light scattering and absorption of metal nanoparticles enable tuning of the excitation wavelength to the NIR region to improve the tissue‐penetration depth of the laser, thereby improving its efficacy in mediating the PDT of tumors in deeper tissues.^109^ Vankayala et al.^106^ reported that gold nanorods (Au NRs) under NIR light excitation could effectively generate ^1^O_2_, thus promoting the death of B16F0 melanoma tumors in mice. By varying the excitation wavelength, it is possible to control whether Au NRs produce ^1^O_2_ or heat, under light irradiation, resulting in tumor cell death by PDT or PTT, respectively (Figure 2A).

Metal oxide nanoparticles, including TiO_2_, ZnO, Fe_2_O_3_, and so on.^109^, ^110^, ^111^ TiO_2_ exhibits photocatalytic ability. When TiO_2_ is irradiated with ultraviolet (UV) light, electrons are excited from the valence band to the conduction band, producing negative electrons (e^−^) while leaving positively charged holes (h^+^) in the valence band. These free electrons react with O_2_ and water molecules in the environment to form O_2_^•−^, H_2_O_2_, and •OH, which in turn react with tumor cell components, leading to apoptosis or necrosis.^112^ Wang et al.^113^ reported an injectable thermosensitive hydrogel (BT‐CTS thermogel) containing magnesium for local tumor treatment and the promotion of wound healing. Magnesium thermal reduction was used to produce structurally defective nanosized black titania (B‐TiO_2_‐*x*) in TiO_2_ nanocrystals. The presence of O_2_ vacancies and disorder‐induced lattice strain in B‐TiO_2_‐*x* enables the light absorption of B‐TiO_2_‐*x* to effectively extend into the visible and NIR regions and produce ROS and heat upon excitation by light. In the presence of B‐TiO_2_‐*x*, which is rich in O_2_ vacancies, the hydrogel showed the dual antitumor effects of PTT and PDT under NIR laser (808 nm) irradiation.

#### 4.2.2 Noble metal complexes

In comparison with conventional organic PSs, noble metal complexes offer numerous advantages in the context of PDT applications. The incorporation of nonessential metals enables the utilization of diverse analytical techniques, such as X‐ray absorption spectroscopy and inductively coupled plasma‐mass spectrometry, facilitating quantitative analysis, and localization of the PSs within cellular and tissue environments. If the metal complex exhibits luminescent characteristics, its precise cellular localization can be further determined through confocal microscopy.^114^ More importantly, noble metal center complexes have received increasing interest as PSs because of their intrinsic heavy‐atom effect, which facilitates strong spin‐orbital coupling and enhances the rate of intersystem crossing. This phenomenon will prolong the time of the triplet excited state (*T*_1_) to interact with dioxygen, biomolecular, or other O_2_ substrates.^115^ Therefore, metal complexes offer a wider range of excited state electronic configurations that can be utilized in both O_2_‐dependent and nondependent cytotoxic pathways.^116^, ^117^, ^118^

The typical metal complexes include Pd(II) complexes,^114^ Ru(II) complexes,^119^ Ir(III) complexes,^98^ and Au(III) complexes.^120^ Mazor et al.^121^ developed WST11 for vascular‐targeted PDT and determined that the inclusion of a Pd center in WST11 enhances stability, excited state reactivity, and intersystem crossing rate of the PS. Fong et al.^122^ developed a Ru(II)‐based agent TLD1433 that is currently undergoing clinical trials for intravesical application in nonmuscle invasive bladder cancers. TLD1433 demonstrated the ability to effectively engage in both type I (electron transfer) and type II (energy transfer) photoreactions, thereby transforming its excited state reactivity from single‐linear O_2_ sensitization in well‐oxygenated environments to photo‐oxidation–reduction reactions under anoxic conditions and displaying ^1^O_2_ quantum yields. Cole et al.^123^ designed several families of Ru(II) bis‐heteroleptic complexes. This new class of light‐responsive, hypoxia‐active agents incorporating the α‐oligothienyl group might involve excited state pathways distinct from the ^1^O_2_ and photosubstitution pathways, as well as a novel mode of electron‐transfer reaction specific to oligothiophenes. They exhibited phototherapeutic indices up to >500,000 in normoxia and >5800 in 1% O_2_ hypoxia. Recently, Mani et al.^124^ reported a series of Os(II) polypyridine complexes as PSs for PDT. As a result of the pronounced π‐extended structure of the ligand and the heavy‐atom effect imparted by the Os center, these complexes demonstrated that heightened absorption in the NIR region increased ^1^O_2_ quantum yields and shifted the maximum wavelength in comparison with their ruthenium analogues.

#### 4.2.3 Carbon nanomaterials

Typical carbon nanomaterials include graphene quantum dot (GQD),^73^, ^125^ C_60_,^126^ and so on. Among them, the absorption spectrum of GQD spans the UV region and the entire visible region, which can be used as a PS for PDT to produce ^1^O_2_ via a multistate sensitization process (Figure 2B).^125^ In addition, C_60_ has unique photophysical properties. Under UV or visible‐light irradiation, C_60_ molecules can be transformed into excited trilinear states, producing single‐linear states or other forms of ROS.^127^ Furthermore, C_60_ is known as a “free radical sponge” and can scavenge ROS by electron transfer in the absence of light, which has the potential to be applied to mitigate the damage caused by the local accumulation of ROS in biological tissues.^128^

#### 4.2.4 Graphene‐like materials

Some graphene‐like materials (e.g., black phosphorus (BP)) can be used as PSs for anticancer.^129^ BP exhibits a thickness‐dependent bandgap ranging from 0.3 eV in the parent material to 2.0 eV in the monolayer, resulting in tunable and broad optical absorption in the UV‐to‐visible range.^130^ Wang et al.^131^ in 2015 first demonstrated that BP nanosheets can efficiently produce ^1^O_2_ with a quantum yield of approximately 0.91, which is higher than that of most reported PSs. In addition, BP‐based nanomedicines have a better photothermal conversion efficiency. Zhang et al.^132^ found that BP‐based nanodrugs can be used in combination with PTT and PDT in cancer treatment through the study of polyethylene glycol (PEG)ylated hyaluronic acid (HA)‐modified BP (HA‐BP) (Figure 2C). In addition, the phototherapy of HA‐BP elicited ICD to mediate antitumor immunity by secreting damage‐associated molecular patterns (DAMPs), including surface‐exposed calreticulin (CRT), adenosine triphosphate (ATP), and high‐mobility group protein B1 (HMGB1), to promote prominent dendritic cell maturation and then induce the activation of specific effector T cells (such as CD4^+^/CD8^+^ T cells) to further eliminate tumor cells.

### 4.3 Molecular mechanisms of organic PSs activation

#### 4.3.1 Tetrapyrrole compounds

**FIGURE 3:**
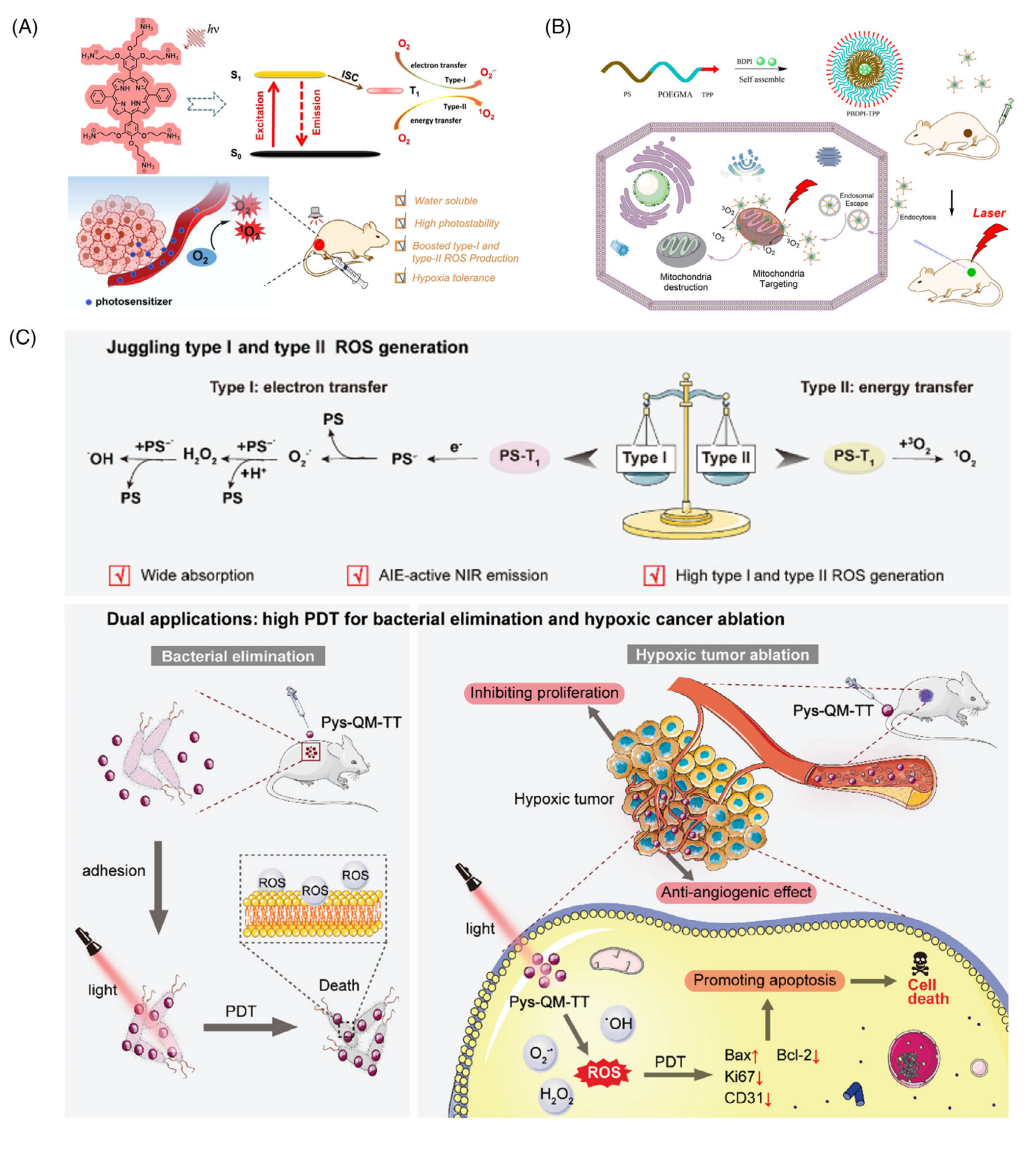
Schematic representation of PDT based on organic materials. (A) TPPN with O‐linked cationic side chains for PDT of hypoxic tumors.^135^ Copyright 2022, American Chemical Society; (B) PBDPI‐TPP nanoparticles for mitochondria‐targeting and imaging‐guided PDT.^138^ Copyright 2020, American Chemical Society; (C) schematic illustration of Pys‐QM‐TT, which produces both type I and type II ROS, for PDT of bacterial infections and tumor upon light irradiation.^154^ Copyright 2023, Wiley‐VCH GmbH. •OH, hydroxyl radicals; ^1^O_2_, singlet oxygen; ^3^O_2_, diradical; AIE, aggregation‐induced emission; Bax, a proapoptotic protein; Bcl‐2, an antiapoptotic protein; CD31, angiogenesis index; H_2_O_2_, hydrogen peroxide; ISC, intersystem crossing; Ki67, a nuclear protein related to cell proliferation; NIR, near‐infrared region; O_2_, oxygen; O_2_^•−^, superoxide anion radicals; PDT, photodynamic therapy; POEGMA, poly‐oligo(ethylene glycol)methacrylate; PS, polystyrene; Pys‐QM‐TT, an electron‐rich anion‐π^+^ AIEgen; ROS, reactive oxygen species; TPP, tetraphenylporphine.

Tetrapyrrole‐type PSs, which mainly include compounds such as porphyrins, phthalocyanines, chlorophylls, and bacteriochlorophylls, are the most commonly used PSs for anticancer therapy. In contrast to PSs with other structures, tetrapyrrole‐type PSs (except bacteriochlorophylls) are primarily type II PSs that produce type II ^1^O_2_ rather than type I ROS.^33^ Tetrapyrrole‐type PSs are unique to PDT because of their high ^1^O_2_ generation efficiency and excellent fluorescence performance.^133^ However, the lack of O_2_ in the TME can seriously affect the therapeutic efficacy of type II PSs. Recently, scientists prepared type I PSs by modifying typical type II PSs to improve the anticancer effects of such PSs.^134^ For example, Sun et al.^135^ prepared porphyrin with O‐linked cationic side chains that exhibited enhanced ROS generation in both type I and type II PDT pathways, and their PDT efficacy under hypoxic conditions was significant (Figure 3A).

#### 4.3.2 Other organic dyes

Organic dyes, such as BODIPY, anthocyanine dyes, and phenothiazine dyes, can also be used as PSs.^136^ Organic dyes such as carbocyanine iodide dyes, which are absorbed in the NIR region, can effectively convert NIR light into fluorescence, ROS, and heat, thus contributing to tumor extinction.^137^ Yuan et al.^138^ used mitochondria‐targeting amphiphilic copolymers to load BODIPY (PBDPI‐TPP) and generated ROS upon laser irradiation, impairing the biological function of organelles and causing apoptosis (Figure 3B). Schneider et al.^139^ also demonstrated the advantageous properties of BODIPY‐based agents in cancer therapy. These agents exhibited high photocytotoxicity and low dark toxicity, with a ratio of dark to light toxicity (phototoxic index) exceeding 830,000 and still exceeding 360,000 under low O_2_ conditions.

#### 4.3.3 CPs

CPs are a class of organic macromolecules characterized by a substantial π‐conjugated backbone, light­harvesting capability, and efficient energy transfer.^140^, ^141^ The electronic conjugation between each repeating unit in their backbone generates a semiconductive “molecular wire,” allowing for efficient migration of excitons along the conjugated backbones upon excitation, thereby enhancing energy transfer efficiency to low‐energy acceptors. Furthermore, it has been reported that encapsulating PSs with CPs into nanoparticles or covalently incorporating them into the main chain of CPs may further enhance their photosensitization efficiency.^142^, ^143^, ^144^ Therefore, CPs‐based PSs have great therapeutic potential for the treatment of malignant tumors. Xing et al.^145^ reported in 2009 the first work utilizing the light‐harvesting CP to amplify ROS generation. They synthesized anionic water‐soluble polythiophene (PTP) and cationic porphyrin (TPPN) to form electrostatic complexes and undergo efficient energy transfer from PTP to TPPN under white light (400–800 nm) irradiation. The polymer PTP could transfer its excited state energy to TPPN via energy transfer, leading to enhanced intersystem crossing in the complex, and the production of TPPN's long‐lived triplet state which further sensitized the ground state O_2_ molecule to produce ^1^O_2_. Zhou et al.^146^ employed a donor‐acceptor strategy to design the backbone structure and synthesized a CP named PTDBD, incorporating electron‐rich thiophene with electron‐deficient benzothiadiazole and diketopyrrolopyrrole. When exposed to an 808 nm laser, PTDBD exhibited efficient conversion of light into heat while generating. Caverzán et al.^147^ constructed CP‐based nanoparticles and demonstrated their ability to generate ROS upon irradiation. Through in vitro and in vivo studies, it was found that the system was capable of inducing direct cancer apoptosis‐involved cell death.

#### 4.3.4 AIE molecules

Most organic PSs, particularly clinically approved porphyrins, have an intrinsic defect that leads to severe intermolecular π–π stacking at high concentrations or in the aggregated state leading to diminished or even complete loss of fluorescence, which is referred to as “Aggregation‐Caused Quenching” (ACQ).^148^ In contrast to conventional PSs, which show quenched fluorescence in the aggregated state and reduced single‐linear O_2_ production, AIE molecules can be excited by light in the aggregated state and emit strong fluorescence while efficiently producing reactive O_2_ species. This new compound overcomes the problem of traditional PSs being prone to fluorescence burst and failure when molecules are aggregated, and thus, it has significant advantages in the field of PDT applications.^149^, ^150^

AIE is a photophysical phenomenon discovered by Luo et al.^151^ in 2001. Owing to the restriction of the intramolecular motion (RIM) of the dye in the aggregated state, the nonradiative leap was suppressed, resulting in a significant enhancement of the fluorescence. Yuan et al.^152^ then developed a kind of AIE NPs with excellent fluorescence imaging and ROS generation capabilities in 2014. Based on the mode of ROS production, AIE‐PSs are mainly classified as type I or type II, and they can generate ROS through both type I and type II pathways.^153^ For example, Wang et al.^154^ recently reported AIEgen Pys‐QM‐TT, which is capable of producing both type I and type II ROS and simultaneous NIR fluorescence imaging for the effective suppression of bacterial infection and ablation of tumor tissue (Figure 3C). In Pys‐QM‐TT, the strong electron‐donating triphenylamine unit, π‐bridge thiophene, and electron‐withdrawing pyridinium salt unit can enhance the D‐π‐A behavior, thereby improving the intramolecular charge transfer effect and extending the emission wavelength. Simultaneously, the strong D‐π‐A effect should reduce π E_S‐T_ and promote intersystem crossing processes, thereby increasing ROS production. In addition, the negatively charged anion in the pyridine salt moiety provides an electron‐rich environment for the excited PS, thus promoting electron transfer to generate type I ROS. In general, the pathway of ROS production by AIEgens is strongly influenced by their structure.

#### 4.3.5 Natural photoactive compounds derived from plants

With the development of separation and extraction techniques, numerous natural photoactive compounds have been isolated.^155^ In recent years, many photoactive compounds such as polyacetylene, thiophene, and anthraquinones have been identified from plant extracts.^156^, ^157^ Some of these compounds are characterized by three‐bonded carbon–carbon molecules and thiophene compounds. Typically, aliphatic compounds conjugated to three or more alkyne bonds are considered phototoxic in nature.^158^ Polyacetylene and thiophene compounds have been reported to be activated or excited, with the polyacetylene compound producing ^1^O_2_ while the thiophene provides high optical yields, resulting in type II PDT reaction yields.^159^, ^160^

Polycyclic quinones PSs are a class of natural PSs derived from mycorrhizal plants, mainly including bamboo red mycorrhizal, curcumin, and chrysin.^161^ Notably, chrysin can efficiently produce single‐linear O_2_ upon laser irradiation, increase superoxide dismutase activity, and decrease cellular glutathione levels.^162^, ^163^ PDT mediated by these compounds can inhibit the proliferation of a variety of tumor cells, including skin cancer,^164^ head and neck squamous cell carcinoma,^165^ breast cancer, and so on.^166^ Due to its hydrophobic nature, chrysin predominantly localizes within lysosomal membranes as well as the Golgi apparatus, endoplasmic reticulum, and mitochondria. Moreover, it exhibits inhibitory effects on tumor cell growth and angiogenesis and induces apoptosis, necrosis, and autophagy of tumor cells.^167^, ^168^

## 5 CURRENT CHALLENGES EXISTING IN THE PDT OF TUMOR

Although PDT has been demonstrated to exhibit tremendous potential as a novel cancer treatment strategy, its clinical application has often been sluggished.^169^ Currently, PDT is only applicable for treating superficial sites of disease or tumors accessible through endoscopic procedures, such as skin cancers and primary tumors.^170^ This section will explore the existing challenges in antitumor PDT, mainly focusing on the three fundamental requirements of PDT (i.e., PS, O_2_, and light).

*PSs*: The nature of PSs plays a critical role in the therapeutic efficacy of PDT. Ideally, suitable PSs for PDT of tumors should possess good water solubility, high stability, strong tumor targeting ability, and negligible dark toxicity to normal tissues.^171^ However, most conventional PSs are small organic molecules, such as porphyrins, phenothiazines, and phenolazines.^169^ Specific groups in the molecular structure of PSs will affect their water solubility. For example, PSs containing multiple aromatic rings, such as porphyrins, exhibit a large number of π‐π stacking interactions between the aromatic rings, resulting in increased intermolecular forces and reduced solubility.^170^, ^171^ PSs containing long‐chain aliphatic groups also have low water solubility due to the weak interaction between aliphatic groups and water. The inadequate solubility of PSs can cause aggregation within physiological environments, which will compromise their stability and hinder effective drug accumulation at the tumor site, ultimately resulting in impaired therapeutic efficiency. Moreover, the excitation wavelengths of the majority of PSs predominantly fall within the UV and visible regions. The restricted depth of tissue penetration poses a potential obstacle to the widespread utilization of PDT.

*O_2_*: The efficacy of PDT is highly dependent on the O_2_ content in the tumor tissue because cytotoxic ROS can only be produced by the energy transfer between activated PS and O_2_ molecules. However, the TME is often in a hypoxic state. Tumor cells exhibit rapid proliferation, heightened metabolic activity, and elevated energy requirements. However, in scenarios where the demand for metabolic O_2_ surpasses its supply, the hypoxic regions within tumor cells expand, thereby diminishing the effectiveness of PDT. The abnormal vascular structure caused by dysregulated angiogenesis is also an important cause of hypoxia. Under hypoxic conditions, the expression of erythropoietic and angiogenic factors will increase, further promoting the proliferation of angiogenic cells, but the formed vessels are nonfunctional, creating a hypoxic environment due to the lack of vascular system in the cancer area. Moreover, PDT can further exacerbate hypoxia, as O_2_ is continuously consumed during the treatment.^172^ Additionally, hypoxia can activate the antioxidant mechanism of tumor cells, rendering them more resistant to PDT.

*Light*: PDT for cancer is currently used both endoscopically to treat superficial tumors and surgically accessible lesions and as an image‐guided adjuvant for the removal of deeper tumors.^173^ It's primarily due to the limited depth of tissue penetration of light. As the lesion deepens, the light energy decays dramatically, resulting in incomplete elimination of tumor cells. Moreover, due to tissue inhomogeneity and heterogeneity, light propagation through the tissue is subject to multiple scattering, further reducing the depth of light penetration. Additionally, different tumor tissues have varying optical properties, such as absorption coefficient, scattering coefficient, and refractive index, which can also impact the propagation and penetration depth of light in the tissues.^174^

Besides, the TME is a highly complex and dynamic ecosystem. The complexity of tumor tissue, including abnormalities in the tumor vasculature, hypoxia, and overexpression of antioxidants, presents biological barriers that can limit the distribution and activity of PSs in tumor tissue and, consequently affect the efficacy of PDT. Additionally, the TME concentrates malignant cells, T cells, B cells, natural killer cells, tumor‐associated macrophages, myeloid‐derived suppressor cells, dendritic cells, tumor‐associated neutrophils, adipocytes, and vascular endothelial cells. The cellular interactions constitute an immune suppressive microenvironment, which can impair the efficacy of PDT‐based photoimmunotherapy.^175^ Many studies also have confirmed that the metabolic, acidic, and neural TME, and the mechanical stress can all have a significant impact on antitumor efficacy.^176^ Therefore, researchers have developed various approaches to address the challenges faced by PDT, among which the development and application of nanocarriers have shown promising results in overcoming many limitations of PDT.

## 6 NANOCARRIERS FOR PDT APPLICATION

In recent years, the field of nanotechnology has seen remarkable advances, especially in the development of nanodrug delivery systems for diagnostic and therapeutic purposes. The use of nanocarriers enables precise delivery of drugs to targeted tissues, leading to increased efficacy of cancer treatments.^177^ When combined with PSs, nanocarriers can significantly improve the efficiency of PDT. First, nanocarriers can alleviate problems associated with PSs, such as poor solubility, potential side effects, and limited tissue penetration. Second, some nanomaterials can address the problem of hypoxia at the tumor site that limits PDT. Moreover, the precise localization of nanocarriers to specific tumor sites can improve the selectivity of PDT.^178^, ^179^ In addition, some nanoparticles are capable of carrying multiple drugs and contrast agents, allowing for the integration of therapy and diagnosis.^180^ It is expected that application of nanocarriers can surmount the challenges of PDT.

### 6.1 Nanocarriers improve water solubility of PSs

It has been reported that 90% of the PSs approved by the US FDA for clinical use are hydrophobic drugs.^181^ The hydrophobic nature makes these PSs easy to aggregate in biological fluids, which greatly impairs the PDT therapeutic efficiency.^182^ To overcome the poor solubility of PSs and improve the efficacy of PDT, various nanocarriers such as organic nanocarriers, inorganic nanocarriers, and organic–inorganic hybrid nanocarriers have been used to create stable dispersions in an aqueous environment to achieve effective delivery of PSs. Organic nanocarriers (liposomes, micelles, polymeric nanoparticles) can be used to solubilize the PS into their hydrophobic cavities for enhanced solubility. Inorganic materials represented by silica, metals, alloys, and various metal compounds can be precisely controlled to form nanostructures (e.g., mesopores or hollow structures) with high specific surface area, thus encapsulating large amounts of PSs to avoid the effects of ACQ. Utilizing the advantages of both organic and inorganic compositions, nanohybrid carriers such as metal‐organic frameworks (MOFs) also can improve the water solubility and biocompatibility of PSs. It should be noted that loading PSs into nanocarriers can avoid the ACQ effect, which is favorable to increase the stability of PSs. Moreover, the utilization of nanocarriers can bring additional features such as tumor targeting and stimulus responsiveness.

**FIGURE 4:**
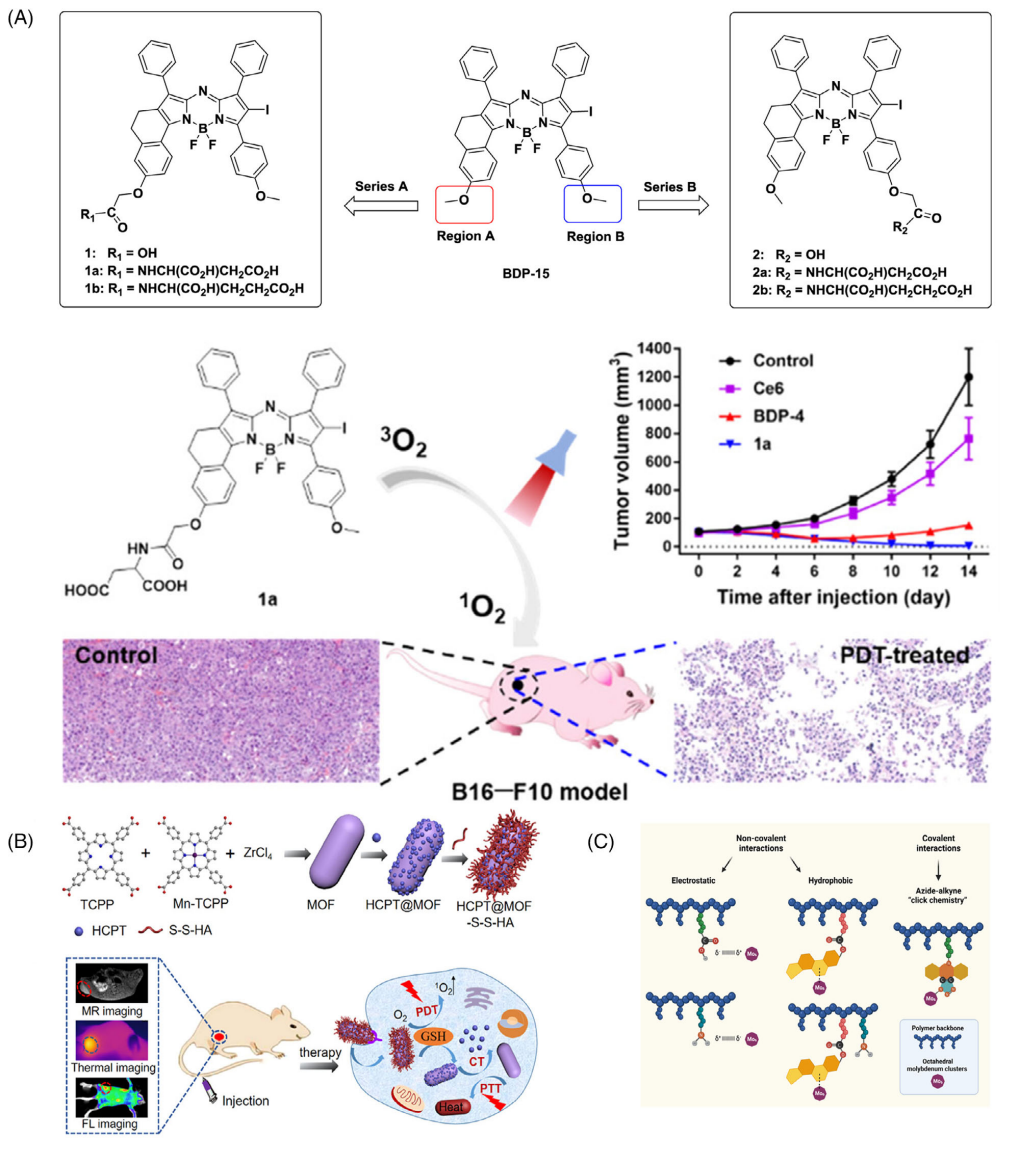
Schematic presentation of nanomaterials to improve water solubility of PSs. (A) Design concept of novel amino acid‐modified aza‐BODIPY PSs. Synthesis and evaluation of aspartic acid modified aza‐BODIPY photosensitizer with higher water solubility and ^1^O*_2_* quantum yield. Reproduced with permission.^184^ Copyright 2022, American Chemical Society. (B) Synthetic scheme of TCPP‐based MOFs for multimodal imaging and enhanced PDT therapy. Reproduced with permission.^185^ Copyright 2022, American Chemical Society. (C) Biocompatible water‐soluble copolymer N‐(2‐hydroxypropyl)methacrylamide (pHPMA) used as carriers for Mo_6_ clusters to improve photosensitizer solubility. Reproduced with permission.^186^ Copyright 2022, MDPI. BDP‐4, 3,5‐di(p‐methoxyphenyl)‐1,7‐diphenyl‐2‐iodo aza‐BODIPY; ^3^O_2_, diradical; BDP‐4, 3,5‐di(p‐methoxyphenyl)‐1,7‐diphenyl‐2‐iodoazo BODIPY; BODIPY, 4,4‐difluoro‐4‐bora‐3a,4a‐diaza‐s‐indacene; ^1^O_2_, singlet oxygen; Ce6, chlorin e6; CT, chemotherapeutic; FL, fluorescence; GSH, glutathione; HCPT, 10‐hydroxycamptothecin; MOF, mixed‐ligand metal‐organic framework; MR, magnetic resonance; PDT, photodynamic therapy; PTT, photothermal therapy; pHPMA, N‐(2‐hydroxypropyl)methacrylamide; TCPP, tetrakis (4‐carboxyphenyl)porphyrin.

An et al.^183^ constructed PS nanoassemblies by self‐assembly of small molecules containing cRGD and disulfide. The reduction of the disulfide bond by a high concentration of glutathione in tumor cells caused the intracellular disassembly of nanoparticles, releasing hydrophobic PS. This system significantly improved the water solubility of the PS, thus increasing the ^1^O_2_ yield. Yu et al.^184^ synthesized a series of za‐BODIPY PS by amino acid modification of the previously reported scaffold of za‐BODIPY, which exhibited higher water solubility, higher ^1^O_2_ generation efficiency, and better light‐to‐dark toxicity ratio. The aspartic acid modified 3,5‐di(p‐methoxyphenyl)‐1,7‐diphenyl‐2‐iodoazo BODIPY (BDP‐4), with intense NIR absorption and high ^1^O*_2_* quantum yield, exhibited a good safety profile and the strongest efficacy against various tumor cell lines (Figure 4A).

Zhang et al.^185^ summarized the current applications and recent advances of tetrakis (4‐carboxyphenyl) porphyrin (TCPP)‐based nanocomposites in PDT therapy of tumors. Besides having the properties of porphyrin PSs, TCPP has a unique advantage: it can form MOFs structures by connecting with metal ions, without requiring additional modification. TCPP‐based MOFs can not only improve water solubility but also maintain TCPP's photosensitizing activity. Additionally, TCPP‐based MOFs can be used as nanocarriers for loading other drugs or for functionalized modifications (Figure 4B).

Octahedral molybdenum (Mo_6_) cluster compounds have been reported as relevant photo/radiosensitizers for PDT and X‐ray induced PDT, which have been intensively investigated for photodynamic applications in the treatment of various diseases. The delivery of Mo_6_ clusters to desired targets, as investigated by Tavares et al.,^186^ may be hampered by their limited solubility and low stability under physiological conditions, thus limiting therapeutic efficacy and increasing side effects. To overcome these obstacles, biocompatible and water‐soluble copolymers based on N‐(2‐hydroxypropyl) methacrylamide (pHPMA) were used as carriers for Mo_6_ (Figure 4C). The hydrodynamic diameter of the covalent polymer cluster conjugates ranged from 7 to 11 nm, and their zeta potential values remained relatively unchanged after 5 days. To further evaluate the photophysical stability, the solutions were monitored over a 5‐day period. The results demonstrated no substantial variations in the emission maxima, quantum yield, and O_2_ quenching constant, confirming the high stability of the photosensitized system in phosphate buffer solution (PBS). The observed stability of the conjugates in PBS highlighted their potential for successful implementation in PDT and underscored their suitability for long‐term photodynamic applications.

### 6.2 Nanocarriers improve PSs’ tissue penetration efficiency

The penetration depth of light is dependent on the wavelength. Light with short wavelengths can hinder deep tissue penetration, while light with excessively long wavelengths results in significant energy absorption by water in tissues. Mainstream PSs are typically excited by light with wavelengths of 600−800 nm, which imposes a limitation on the penetration depth within biological tissues. As a result, PDT is primarily utilized for treating superficial diseases in clinical settings. Nevertheless, the application of PDT to deep‐seated tumor tissues remains a formidable technical obstacle that necessitates significant advancements in both basic research and clinical practice. Nanomedicines can be utilized in various approaches to enhance the tissue penetration depth of PSs.^187^ One approach is to leverage the optical properties of nanodrugs to convert low‐energy, long‐wavelength light into high‐energy, short‐wavelength light, such as UCNPs^188^ and two‐photon excitation nanodrugs.^189^ Another approach involves using nanoscintillators to convert X‐rays into visible light, which in turn activates surrounding PSs, enabling PDT of deep tissues.^187^ The third approach requires using visible light generated by high‐speed charged particles traveling through the medium to activate PSs in the surrounding area. Last, bioluminescent energy transfer systems can be employed to produce light sources inside the organism to excite the PS. These approaches have the potential to enable nanomedicines to act as PDT in deep tissues with improved therapeutic efficiency.

#### 6.2.1 Harnessing the optical properties of nanomedicines

In recent years, UCNPs have emerged as a highly promising tool for cancer therapy. UCNPs usually consist of a crystalline matrix doped with lanthanide, transition metal, or actinide ions, which exhibit the unique ability to emit higher‐energy photons by absorbing two or more low‐energy NIR photons and converting them energetically.^190^ This property allows UCNPs to emit light in the UV, visible, or NIR regions, facilitating ultrasensitive immunoassays, high‐contrast imaging, and deep tissue imaging with minimal optical background. To enhance their therapeutic potential, PSs can be conjugated to UCNPs using various methods such as surface modification, chemical bonding, or encapsulation.^191^ These approaches leverage the upconversion luminescence properties of UCNPs and combine them with those of PSs, thereby improving drug delivery efficiency and therapeutic efficacy. Furthermore, the upconversion luminescence properties of UCNPs can enable precise tumor imaging and localization, providing accurate guidance for treatment. This integration of UCNPs and PSs holds great promise in advancing cancer therapy by offering enhanced imaging capabilities and improved treatment precision.^192^

**FIGURE 5:**
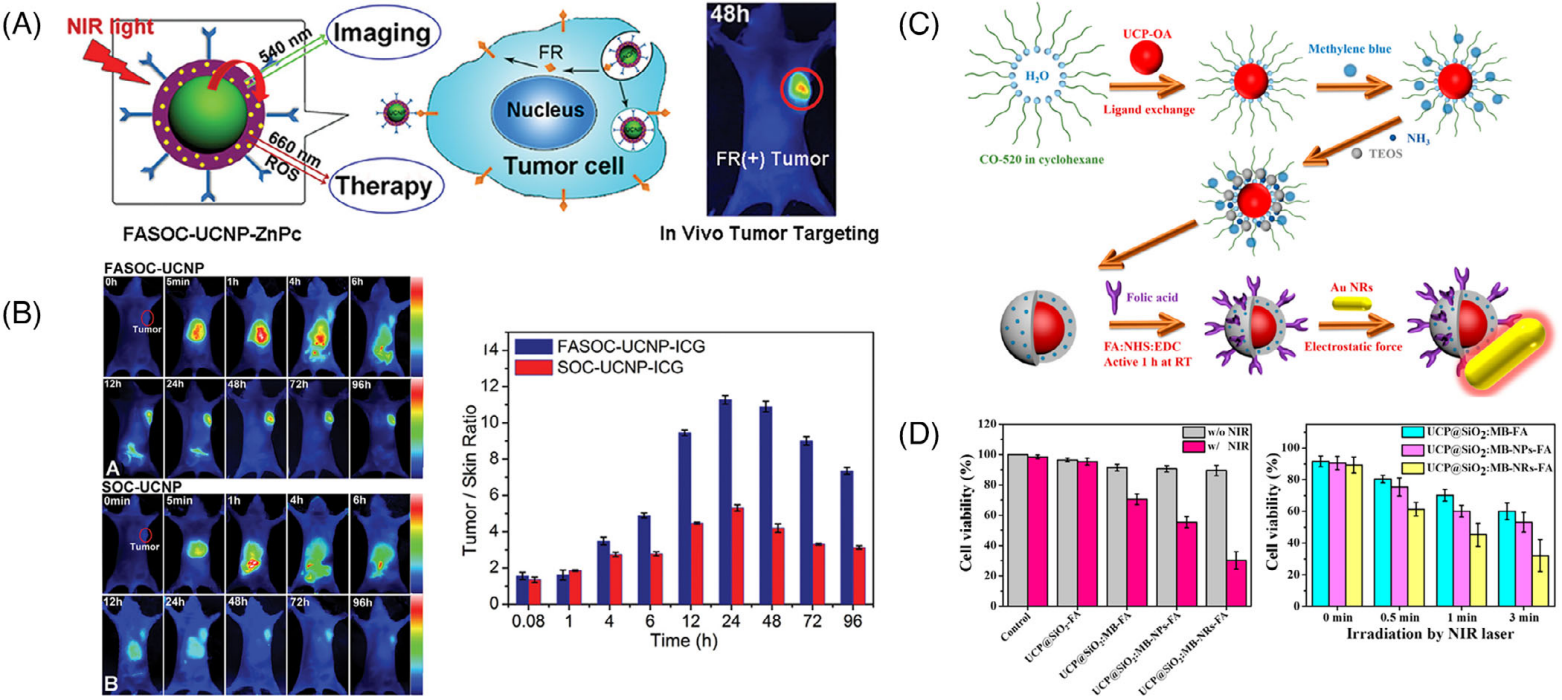
Schematic presentation for harnessing the optical properties of nanomedicines to improve PSs’ tissue penetration efficiency. (A) Schematic illustration of the synthesis of NIR triggered upconversion nanoconstructs for targeted deep‐tissue PDT therapy. Reproduced with permission.^193^ Copyright 2013, American Chemical Society. (B) The in vivo fluorescence images and tumor/skin ratio of tumor‐bearing nude mice injected with different nanoconstructs. Reproduced with permission.^193^ Copyright 2013, American Chemical Society. (C) Schematic diagram of the preparation of upconversion nanoparticle UCP@SiO_2_: methylene blue ‐Au NRs. The silica shell was coated with reverse‐phase microemulsion and modified with folic acid and Au NRs through NHS/EDC coupling reaction and electrostatic force, respectively. Reproduced with permission.^199^ Copyright 2016, American Chemical Society. (D) PDT efficacy investigated by relative cell viability test and time‐dependent irradiation influence.^199^ Copyright 2016, American Chemical Society. Au NR, gold nanorods; FA, folic acid; FASOC, folate‐modified amphiphilic chitosan; FR, folate receptor; ICG, indocyanine green; MB, methylene blue; NIR, near‐infrared region; ROS, reactive oxygen species; UCNP, upconversion nanoparticle; UCP, upconversion nanoparticle; ZnPc, zinc(II) phthalocyanine.

For example, Cui et al.^193^ reported the application of UCNPs in PDT by developing a multifunctional PDT PS. UCNPs were first coated with a porous silica layer that contained high absorbance photosensitive molecules to match the emission spectrum of UCNPs. Specific antibodies were then covalently attached to the silica‐coated nanoparticles to recognize specific antigens expressed on the surface of targeted cells. When irradiated with infrared light, the UCNPs emitted light that was absorbed by the photosensitive molecules on their surface. The excited photosensitive molecules interacted with the surrounding ground state molecular O_2_ to generate singlet O_2_, which led to oxidative damage of the cancer cells. A multifunctional nanostructure consisting of UCNPs and the PS zinc(II) phthalocyanine (ZnPc) has also been developed for the conversion of NIR light into visible light (Figures 5A and B). The surface of UCNPs was coated with folic acid‐modified amphiphilic chitosan to anchor ZnPc close to the UCNPs, facilitating resonance energy transfer from UCNPs to ZnPc. In vivo anti‐S180 tumor effect showed that this nanostructure‐based NIR light‐triggered PDT had significant therapeutic effects.

Two‐photon absorption (TPA) is a nonlinear optical effect that offers several advantages over single‐photon absorption, including the ability to achieve localized photoactivation in deep tissues using higher optical densities and longer wavelengths.^33^ TPA can be used in PDT to selectively irradiate tissues at different depths by selecting the laser wavelength, and it can also improve the efficiency of PS activation and enhance the therapeutic effect.^194^ Moreover, TPA can realize high‐resolution 3D microscopic imaging in deep tissues due to its ability to achieve local excitation with a large laser beam diameter.

Currently, two‐photon excited nanomedicines have been investigated based on various materials, including quantum dots,^195^ Au nanomaterials,^196^ silicon nanomaterials, carbon nanomaterials, and polymer nanomaterials. For instance, Secret et al.^197^ developed a novel PS carrier mannitol‐modified porous silica (pSiNPs). pSiNPs could absorb two‐photons and act as an energy transfer donor to activate the PS by two‐photons at 800 nm, thereby improving the therapeutic effect of PDT. The porphyrin loaded pSiNP nanodrug had a TPA cross section six times higher than that of porphyrin, which showed significant inhibitory effects on MCF‐7 cells, suggesting that two‐photon‐excited nanodrugs could enhance the therapeutic effect of PDT.

Au nanoparticles are also being widely used in two‐photon excited nanomedicines due to their excellent light scattering and absorbing properties. Their extremely high cross section or TPA cross section under two‐photon excitation allows for deeper tissue penetration and more precise control compared with conventional single‐photon absorption.^198^ Chen et al.^199^ conducted a study focusing on the enhancement of PDT efficacy using a nanocomposite composed of NaYF4:Yb/Er UCNPs conjugated with Au NRs. To achieve plasma‐enhanced PDT, methylene blue was encapsulated within silica shells. The UCPs functioned as photoconverters, converting NIR light to visible light to excite methylene blue and generate ROS. The presence of Au NRs contributed to enhance upconversion efficiency and ROS production through the localized surface plasmon resonance effect. The researchers investigated the optimization of methylene blue loading, ROS generation capacity, and effective distance for plasma‐enhanced ROS generation by adjusting the thickness of the silica shell. The mechanism of plasma‐enhanced PDT was further elucidated by enhancing the upconversion luminescence intensity through the plasma field, thereby improving the light‐harvesting ability and absorption cross‐section of the system (Figures 5C and D). Comparative studies involving different surface plasmon resonance bands of Au nanoparticles confirmed the improved ROS generation. In vitro and in vivo experiments demonstrated that the nanocomposite exhibited substantial ROS generation and efficient PDT treatment.

#### 6.2.2 Conversion of X‐rays into visible light using nanoscintillators

**FIGURE 6:**
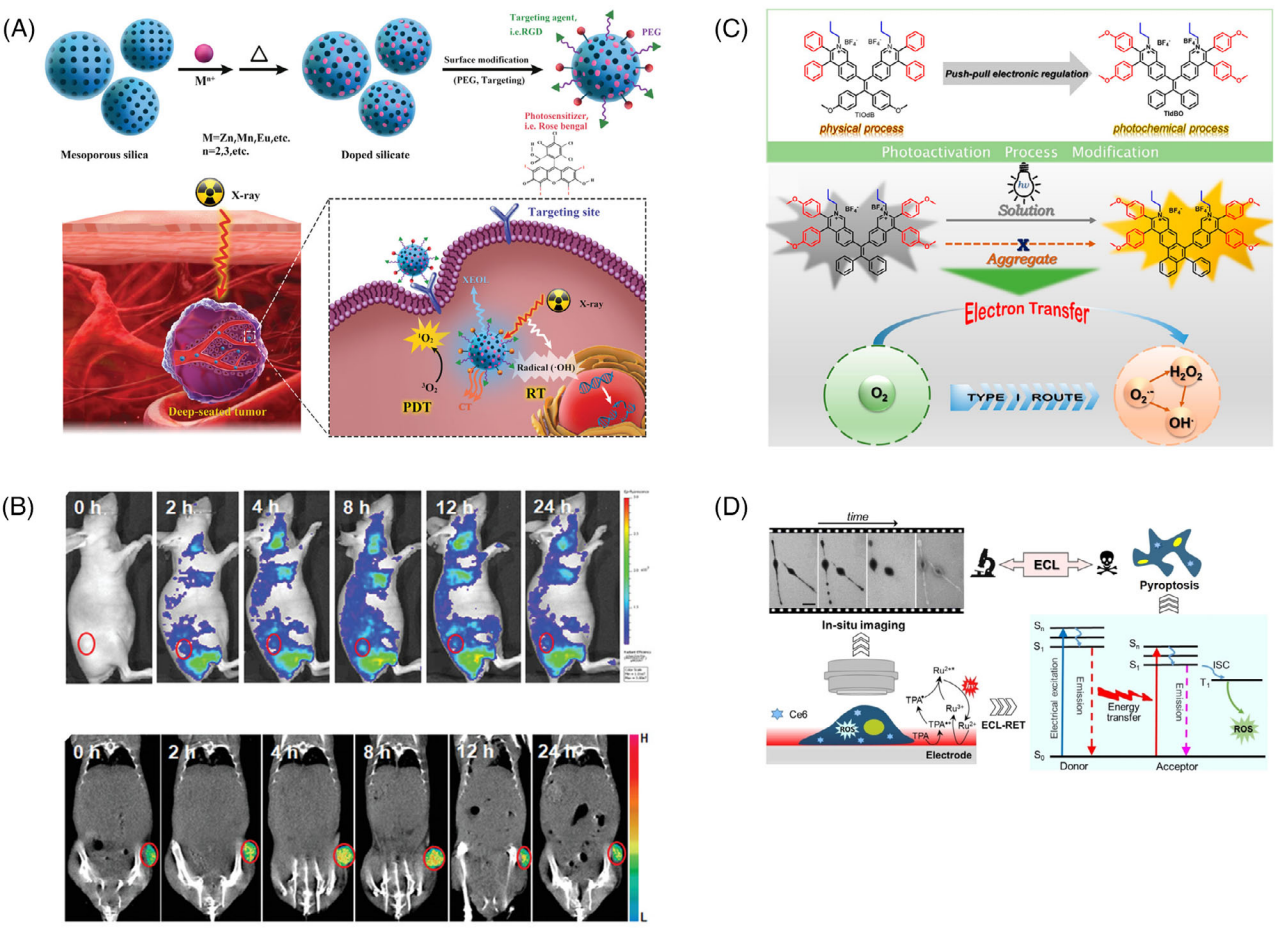
Schematic presentation of nanomaterials to improve PSs’ tissue penetration efficiency. (A) Schematic illustration of the preparation process of mesoporous silicate nanosensitizers and the mechanism for X‐ray‐induced deep‐penetrating PDT therapy. Reproduced with permission.^200^ Copyright 2019, Wiley. (B) Corresponding fluorescence intensities and CT signals of tumor‐bearing mice after intravenously injected with nanosensitizers at 2, 4, 8, 12, and 24 h postinjection. Reproduced with permission.^200^ Copyright 2019, Wiley. (C) Schematic illustration of the molecular structure of TIOdB and its working mechanism for enhanced type I ROS‐generating ability. Reproduced with permission.^201^ Copyright 2021, American Chemical Society. (D) Schematic representation of in situ imaging of the ECL‐PDT process at the level of single living cells. Reproduced with permission.^202^ Copyright 2022, Wiley. ECL‐RET, electrochemiluminescence resonance energy transfer; ECL, electrochemiluminescence; PDT, photodynamic therapy; PEG, polyethylene glycol; RGD, arginylglycylaspartic acid; RT, radiation therapy; TIdBO, an isoquinolinium organic salt derivative based on the TPE skeleton; TPA, tripropylamine; XEOL, X‐ray‐excited optical luminescence.

To harness the tissue‐penetrating power of X‐rays for PDT, researchers often use scintillators with wide energy bandgaps to absorb X‐rays and convert them into UV or visible light, which then excite PSs to produce ROS and achieve therapeutic effects. In a recent study, Chen and colleagues proposed a novel nanomaterial for X‐ray‐based PDT.^200^ This material was composed of a silicate nanoscintillator containing zinc, manganese, and bengal red (Figure 6A). After incubating these nanoparticles with U87MG cells, significant cancer cell death was observed only under X‐ray irradiation. The researchers then intravenously injected the nanoparticles into tumor‐bearing BALB/c nude mice and irradiated them with X‐rays, leading to a remarkable tumor suppression rate of 98.1% compared with the control group. Notably, this silicate nanoscintillator could accumulate in tumors and significantly inhibit tumor progression at low doses of X‐ray irradiation with minimal effects on normal tissues (Figure 6B). These findings suggest the potential of X‐ray‐based PDT in cancer treatment.

#### 6.2.3 High‐speed charged particles activate PSs

In 1934, the Soviet physicist Pavel A. Cherenkov (1904–1990) made a remarkable discovery, observing that high‐velocity charged particles traveling through a medium emitted a faint blue visible light. This phenomenon, known as Cherenkov radiation, can be attributed to various processes such as external light sources, Compton γ‐ray scattering, or the photoelectric effect. Cherenkov radiation has proven to be an effective approach for realizing free‐electron laser light sources. Inspired by this, researchers have explored the potential of utilizing the visible light emitted when high‐velocity charged particles traverse a medium to activate PSs in the surrounding area. In a study, Tang's team developed an isoquinoline organic salt derivative, TIdBO, based on a tetraphenylethylene backbone as a PS (Figure 6C). Under continuous light, small molecules underwent a photocyclization reaction via electron transfer involving free radicals. However, in aggregated form, the photocyclization reaction was suppressed, resulting in a higher proportion of type I ROS products. This demonstrated the feasibility of using molecular aggregation to regulate the competition between the two processes and enhance type I ROS production. Notably, TIdBO not only showed good PDT performance during the interaction with HeLa cells but also achieved self‐monitoring of the PDT process through the relationship between the increase in fluorescence intensity and the change in cell morphology as an indicator of apoptosis.^201^

#### 6.2.4 Bioluminescence light source to activate PSs

Conventional PDT uses external light to irradiate tissues, which face the problems of poor penetration and rapid attenuation. To overcome these problems, researchers are actively exploring internal light sources for self‐luminescence, including chemiluminescence and bioluminescence. However, the intensity and area of self‐luminescence are difficult to control, which limits its clinical application. Therefore, it is important to develop a highly efficient and spatiotemporally controllable self‐luminous PDT system. Tang's team reported a PDT system driven by electrochemiluminescence (ECL). The luminescence generated by [Ru(bpy)_3_]^2+^ and the coreactant tripropylamine pair was both an optical readout for ECL imaging and a light source for excitation of the PS chlorin e6 (Ce6). This system relied on the effective energy transfer from ECL emission to the PS, which excited the surrounding O_2_ for PDT. The dynamic processes of gradual morphological changes, changes in cell‐matrix adhesion, and increases in cell membrane permeability during ECL‐PDT were monitored with good spatial and temporal resolution under ECL microscopy (Figure 6D). It was expected to assist tumor treatment and imaging to reach deeper tumor sites for subsequent treatment.^202^

### 6.3 Nanocarriers enhance the tumor‐specific delivery of PSs

#### 6.3.1 Passive targeting of PSs to tumors

**FIGURE 7:**
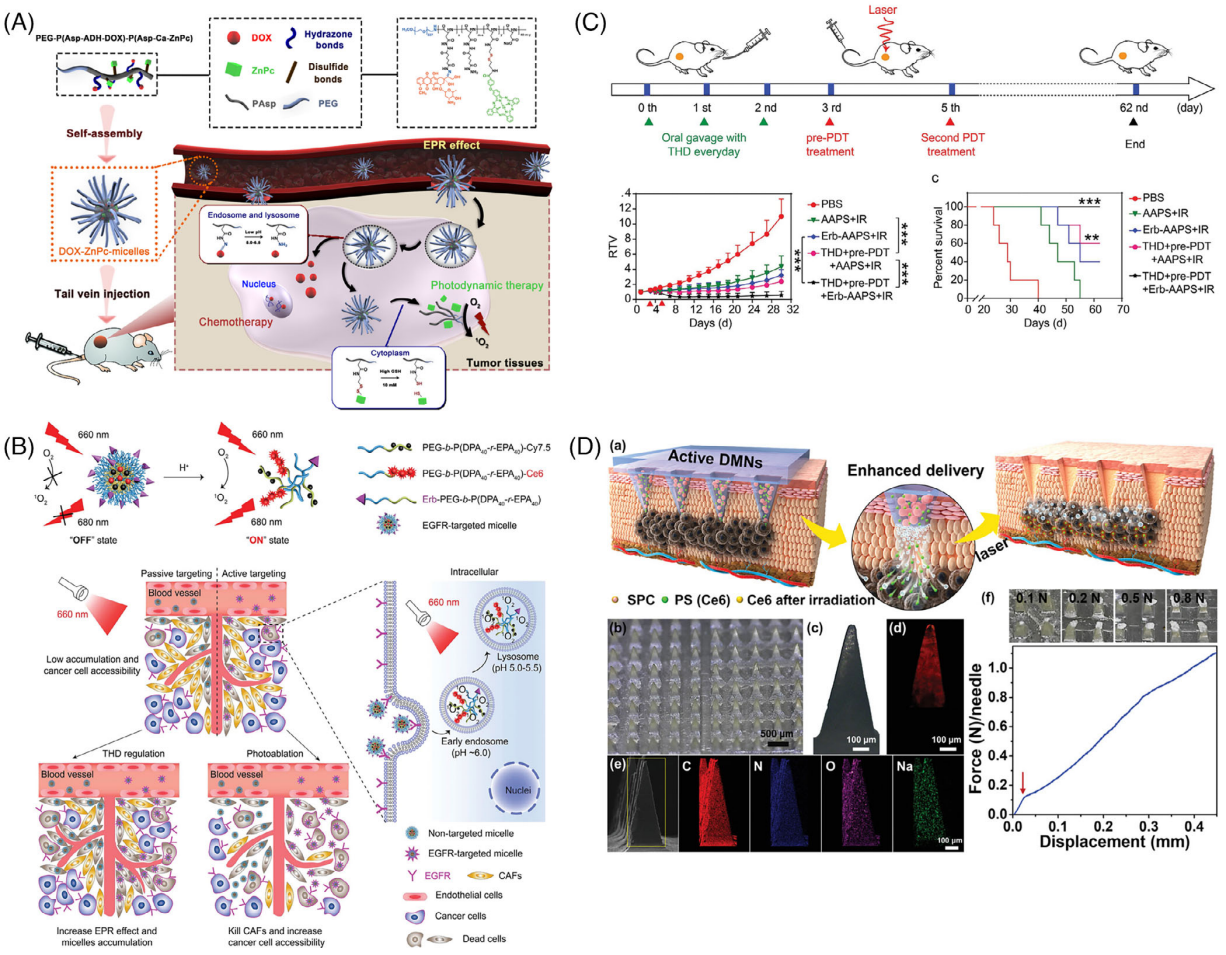
Schematic presentation of nanomaterials to enhance the tumor‐specific delivery of PSs. (A) Schematic illustration of the structure of the pH‐ and redox‐responsive prodrug DOX–ZnPc–micelles and their action mechanism for chemotherapy and PDT therapy. Reproduced with permission.^205^ Copyright 2018, Elsevier. (B) Schematic diagram showing the delivery of acid‐activatable EGFR‐targeted nanophotosensitizer after modulations of the TME. Reproduced with permission.^207^ Copyright 2021, National Library of Medicine. (C) Combined tumor regulations synergistically amplified active targeting and therapeutic efficacy. Reproduced with permission.^207^ Copyright 2021, National Library of Medicine. (D) Schematic illustration of the Ce6‐loaded active DMNs for enhanced transdermal delivery of photosensitizer, and a series of characterizations of Ce6‐loaded active DMNs. Reproduced with permission.^211^ Copyright 2022, National Library of Medicine. AAPS, acid‐activatable nanophotosensitizer; Asp, aspartate; CAFs, cancer‐associated fibroblasts; Ce6, chlorin e6; DOX, doxorubicin; EGFR, epidermal growth factor receptor; EPR, electron paramagnetic resonance; Erb, erbitux; IR, infrared radiation; MN, microneedle; PBS, phosphate buffer solution; PDT, photodynamic therapy; PEG, polyethylene glycol; SPC, sodium percarbonate; THD, thalidomide; ZnPc, zinc(II) phthalocyanine.

The traditional enhanced permeability and retention (EPR) effect allows substances to preferentially infiltrate and remain in tumor tissues compared with normal tissues.^203^ This effect arises from the rapid and disorderly proliferation of tumor vessels, leading to structurally incomplete vessels with large gaps, and the lack of lymphatic vessels further contributes to the EPR effect. This phenomenon facilitates the accumulation of nanoparticles in tumor tissues for passive targeted therapy.^204^ To optimize the passive targeting efficiency, nanoparticles’ physicochemical properties, including size, charge, and shape, should be optimized. Strategies to enhance the EPR effect also include using external physical stimuli (e.g., radiation or heat) to temporarily increase tumor tissue permeability or employing drug administration to disrupt the TME and enhance nanoparticle accumulation. For instance, Gao et al.^205^ developed polymeric micelles containing the chemotherapeutic agent doxorubicin (DOX) and PS ZnPc. These micelles were self‐assembled from amphiphilic block copolymers of methoxy‐PEG and poly(β‐benzyl‐l‐aspartate), which were expected to accumulate in tumor tissue through the EPR effect. The photoactivity of ZnPc was initially inhibited within the micelles due to self‐aggregation. After internalization by tumor cells, the acidic and reducing intracellular conditions would trigger the release of both DOX and ZnPc, resulting in dual therapeutic effects (Figure 7A). To investigate the biodistribution of the DOX–ZnPc micelles, nude mice with HepG2 tumors were treated with an intravenous dose of these micelles. Fluorescence imaging revealed minimal fluorescence in the first 8 h, indicating ZnPc remained within the micelles with strong self‐quenching. Over the next 7 days, fluorescence gradually increased, demonstrating preferential accumulation of DOX–ZnPc micelles at tumor sites through the EPR effect and successful release of ZnPc at the target site. Moreover, Ikeda‐Imafuku et al.^206^ devised a potent anticancer strategy in which DOX was conjugated to PEG through a thioketal linker sensitive to ROS. The obtained amphiphilic PEG–DOX conjugate was used as the nanoparticle carrier for hydrophobic PS pheophorbide A. This system harnessed the EPR effect to augment nanoparticle accumulation within tumor tissues. This treatment approach not only activated pheophorbide A to generate cytotoxic ROS, but also initiated an ROS cascade, inducing structural disruption of the nanoparticles and facilitating the accelerated liberation of DOX and pheophorbide A. As a result, this approach significantly amplified the therapeutic efficacy of the anticancer treatment.

#### 6.3.2 Active targeting of PSs to tumors

Recent investigations have unveiled that nanoparticles can primarily infiltrate into tumors via receptor‐mediated cytosolic transport. Therefore, tailoring nanoparticles for binding to specific receptors on target cell surfaces has shown considerable promise in enhancing targeting precision. Consequently, it may be more productive to pivot research emphasis from EPR‐mediated passive targeting to the strategic design of actively targeted nanotherapeutics. These systems can elevate drug concentrations within target cells, facilitated by the interaction between surface‐bound ligands and membrane receptors on the target cells. A variety of ligands, including antibodies, antibody fragments, or short peptides, have been integrated into nanocarriers with remarkable specificity and affinity. For example, Yan et al.^207^ developed an epidermal growth factor receptor (EGFR)‐targeted hyperpH‐sensitive nano‐PS and systematically evaluated its active targeting ability and therapeutic efficacy after modulation of the tumor vasculature system and stromal barrier (Figure 7B). EGFR, one of the most common targets for cancer therapy, has been reported to be overexpressed on the surface of a variety of cancer cells. And the Fab' fragment of Erbitux, a clinically used antibody against EGFR, was coupled to the nano‐PS as a targeting moiety. The study demonstrated that antibody‐modified nano‐PSs exhibited specific targeting ability and superior cytotoxicity in vitro compared with their nontargeted counterparts. However, in vivo tumor targeting and inhibition were compromised due to stromal cell accumulation and nonspecific isolation. To improve the in vivo behavior of antibody‐modified nano‐PSs, the TME was perturbed (Figure 7C). The synergistic enhancement of tumor accumulation and targeting ability of antibody‐modified nano‐PSs was achieved by sequential modulation of the TME using thalidomide and PDT pretreatment, further highlighting the superiority of the active targeting strategy.

Although this active targeting approach allows for targeted delivery of PSs, incidental uptake of these couplers by normal cells is still unavoidable because most cancer‐associated receptors are not exclusively expressed in cancer cells. To circumvent these problems, dual receptor targeting strategies have been explored to facilitate targeted delivery of photosensitizing drugs. Since cancer cells often overexpress more than one surface receptor, this dual‐receptor targeting strategy can increase the likelihood of ligand–receptor interactions, resulting in better uptake and more precise targeting of cancer cells compared with single‐receptor targeting approaches. Ng's group has devised a novel strategy for activating such a PS specifically in the target cells via dual receptor‐mediated bioorthogonal coupling. This system consists of two components, a biotin‐modified tetrazine‐substituted PS and a cyclic EGFR‐targeting peptide linked with a bicycle[6.1.0]non‐4‐yne dienophile, which are preferentially internalized only by cancer cells expressing both biotin receptors and EGFR, followed by a rapid bio‐orthogonal inverse electron‐demand Diels‐Alder reaction to form the corresponding cycloadduct, thereby restoring the PS's ability to fluoresce and generate ROS.^208^

Due to the good in vitro targeting behavior of actively targeted nanomedicines, their related studies are increasing year by year. However, in vivo, the complex TME often affects the targeting effect of actively targeted nanoparticles. It was reported that after intravenous injection of nanoparticles, only a very small amount of particles can be delivered to solid tumors by the EPR effect.^209^ In addition, after leaving the tumor vasculature, actively targeted nanoparticles must traverse a long interstitial pathway to reach target cells. During this process, several physiological barriers will limit their penetration from the tumor vasculature into deeper tumor tissues, such as spatial blockage of the extracellular matrix and nonspecific uptake by tumor‐associated fibroblasts and tumor‐associated macrophages. These factors severely limit the delivery efficiency and efficacy of actively targeted nanomedicines. Therefore, the therapeutic efficacy of active targeting is still controversial.

#### 6.3.3 Localized delivery of PS

Localized administration of PSs holds significant promise for treating superficial tumors while minimizing systemic phototoxicity. Yet, the efficacious migration of PSs into targeted tumor tissues is challenging due to the stratum corneum barrier.^162^, ^210^ In this context, dissolving microneedles has emerged as a promising solution, exhibiting distinct advantages to surmount the stratum corneum through creating several mechanical channels across the skin. Particularly in the case of superficial tumors, dissolving microneedles offer direct nanomedicine delivery to superficial lesion sites, effectively circumventing the uneven dispersion and potential toxicity inherent to systemic drug administration. Furthermore, dissolving microneedles can uphold PS stability and effectiveness during storage and transport, thereby addressing pivotal requisites in PDT.^164^ Liu et al.^211^ introduced a novel approach using PS‐loaded dissolving microneedle patches armed with O_2_ propellant [referred to as PS‐loaded active dissolving microneedle patches (DMNs)] to actively transport PS to tumor sites (Figure 7D). Constructed from polyvinylpyrrolidone‐solubilized polymers infused with Ce6 and sodium percarbonate particles, the PS‐loaded active DMNs could achieve precise and efficient drug delivery to tumors. Upon skin insertion, the embedded sodium percarbonate particles instantaneously reacted with the adjacent dermal mesenchyme to generate gaseous O_2_ bubbles, which could enhance PS penetration depth while mitigating tumor hypoxia during laser irradiation, thereby amplifying PDT effectiveness. Notably, in vivo assessments using tumor‐bearing mouse models exhibited a significant inhibition of tumor growth and a 50% increase in survival through active delivery. This investigation offers an expedient and efficient avenue for PS delivery, underscoring the potential of dissolving microneedle drug delivery as a novel strategy for advancing PDT of tumors. In another research, Luo et al.^212^ ingeniously combined chemo‐phototherapy with microneedle drug delivery, employing MIL‐100(Fe) nanoparticles as a carrier to codeliver the hydrophobic PS ZnPc and the chemotherapeutic agent DOX. By encapsulating the nanoparticles into microneedles for direct tumor delivery without systemic circulation, the concentration of ZnPc at the tumor site was elevated, while the potential cardiac toxicity of DOX was mitigated. A series of in vitro and in vivo investigations confirmed the synergistic potential of chemo‐phototherapy in curtailing tumor progression. Consequently, the nanoparticle integrated microneedle patch, as pioneered in these studies, holds considerable promise as a pivotal benchmark in the realm of PDT for superficial tumors.

### 6.4 Nanocarriers enhance the PDT efficacy in hypoxic environments

The therapeutic efficacy of PDT heavily relies on the O_2_ content in tumor tissues. However, tumors often suffer a hypoxic microenvironment due to their rapid cell proliferation and insufficient blood supply,^172^ which greatly limits the effectiveness of PDT. Moreover, the rapid consumption of O_2_ during PDT further exacerbates tumor tissue hypoxia. Hence, there is a pressing need to continuously explore new approaches to enhance the efficacy of PDT in hypoxic environments.^213^ Studies have reported several methods that can ameliorate the hypoxic microenvironment at the tumor site, including: (1) O_2_ delivery via O_2_ carriers^214^; (2) direct production of O_2_ within tumor tissue^215^; (3) improvement of tumor tissue perfusion by heating; and (4) remodeling the TME by degrading the extracellular matrix.

**FIGURE 8:**
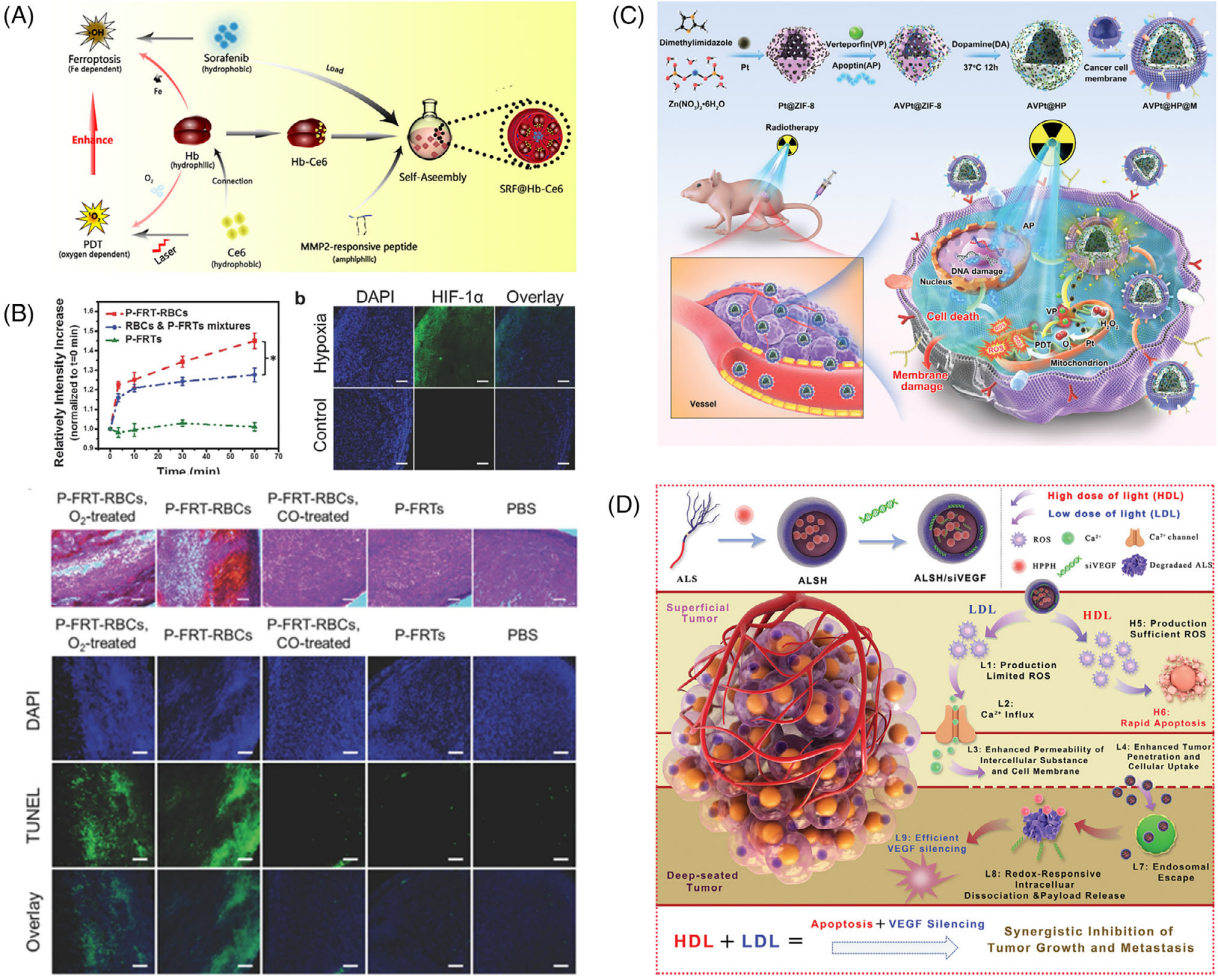
Schematic presentation of nanomaterials to enhance the PDT efficacy in hypoxic environments. (A) Schematic illustration showing the construction of a two‐in‐one nanoplatform by linking hemoglobin to photosensitizer Ce6, followed by loading with sorafenib (ferroptosis promoter) for combining O_2_‐amplified PDT and potent ferroptosis. Reproduced with permission.^216^ Copyright 2020, American Chemical Society. (B) The conjugation of RBCs with photosensitizer encapsulated nanocapsules showed an enhanced PDT effect under hypoxic environments. Reproduced with permission.^217^ Copyright 2016, National Library of Medicine. (C) Schematic illustration of biomimetic nanoplatform based on hollow polydopamine nanoparticles and multiple strategies to sensitize radiotherapy of colon cancer. Reproduced with permission.^219^ Copyright 2022, Wiley. (D) Schematic illustration showing the construction of an alternating radiation curation‐driven PDT and RNA interference combination therapy for the successful inhibition of tumor growth and metastasis. Reproduced with permission.^60^ Copyright 2022, Wiley. ALS, cationic amphipathic peptide; ALSH, HPPH‐loaded ALS nanoparticles; AP, apoptosis; DA, dopamine; DAPI, 4,6‐diamidino 2‐phenyl‐indole dihydrochloride; Hb, hemoglobin; HDL, high dose of light; HIF‐1ɑ, hypoxia‐inducible factor 1; HPPH, codelivery system of photochlor; LDL, low dose of light; P‐FRT, ferritin; PBS, phosphate buffer solution; PDT, photodynamic therapy; RBC, red blood cell; RT, radiation therapy; SRF, sorafenib; VEGF, vascular endothelial growth factor; VP, verteporfin; ZIF‐8, zeolitic imidazolate framework.

Recent advances in nanocarriers have opened up great prospects for the development of new PDT systems. On the one hand, exogenous molecular O_2_ can be captured by biological, biomimetic, or physical mechanisms and then transported to solid tumors using nanocarriers. On the other hand, O_2_ can be generated in vivo by PDT‐related materials or other methods to provide a continuous local O_2_ supply to improve PDT efficiency. With the emergence of Fe‐dependent ferroptosis boasting ROS cytotoxicity as well, such a chemodynamic approach to cancer therapy has drawn extensive attention. Xu et al.^216^ attached hemoglobin to the PS Ce6 to construct a two‐in‐one nanoplatform loaded with sorafenib, an iron oxidation promoter, thus combining O_2_‐assisted PDT with potent iron oxidation therapy (Figure 8A). Since hemoglobin itself contains iron that binds to O_2_, it can simultaneously provide O_2_ for O_2_‐dependent PDT and iron for iron‐dependent iron oxidation. The in vitro and in vivo experiments showed that this system could remarkably enhance PDT efficiency.

Red blood cells (RBCs) can be used as carriers for both PS and O_2_ since each RBC can carry 270 million hemoglobin molecules (each hemoglobin binds four O_2_ molecules). The long circulating half‐life and minimal extravasation of RBCs are additional advantages for PDT, ensuring the greatest possible photodynamic impact on the lumen of the tumor vasculature and endothelium. However, most PSs are porphyrin‐like molecules that are hydrophobic, which do not provide functional groups to facilitate conjugation. This problem can be solved by using nanocapsules to encapsulate the PS and further bind the conjugate to the RBCs surface. Recent studies by Xie et al. showed that ferritin could be loaded with 40 wt% of PS such as ZnF16 Pc without inducing significant self‐quenching or affecting colloidal stability by biotin‐neutrophil protein‐mediated coupling. Then the ZnF16Pc‐loaded ferritin was conjugated to RBCs. The resulting conjugates carried large amounts of PS and O_2_ and efficiently produced ^1^O_2_ even under hypoxic conditions. A series of in vitro and in vivo experiments were conducted in hypoxic tumor models, and the results suggested that RBC‐PDT had great potential in cancer therapy, which provides a new avenue for improving the efficacy of PDT^217^ (Figure 8B).

In contrast to normal cells, tumor cells exhibit a heightened accumulation of H_2_O_2_, previously attributed to the generation of superoxide anions (O_2_^−^) from cytosolic nicotinamide adenine dinucleotide phosphate (NADPH) oxidase or the mitochondrial respiratory chain, alongside the action of the superoxide dismutase enzyme.^218^

The unveiling of the mammalian NADPH oxidase family in recent years has revitalized the understanding of H_2_O_2_ production mechanisms within tumor cells. This contemporary perspective posits that H_2_O_2_ generation occurs at diverse cellular locales, encompassing the cell membrane, mitochondria, peroxisomes, and conceivably other as‐yet‐unidentified sites. Consequently, a promising avenue emerges wherein the conversion of endogenous H_2_O_2_ to O_2_ can serve as a countermeasure against hypoxia induced by PDT. In addition to catalyzing the degradation of H_2_O_2_ to H_2_O and O_2_, many studies have focused on the codelivery of peroxidase and PS to tumor tissue. Gong et al.^219^ designed a bionanoparticle‐based platform consisting of hollow polydopamine bounded with Pt nanoparticles, which has peroxidase‐like activity, to trigger endogenous H_2_O_2_ to O_2_ and alleviate hypoxia in the TME (Figure 8C). This nanosystem exerted radiosensitizing effects through multiple strategies, including alleviation of hypoxia, enhancement of tumor apoptosis, and X‐ray induced PDT.

PDT‐induced hypoxia in the TME can lead to the overexpression of several vascular growth factors, such as vascular endothelial growth factor (VEGF) and cyclooxygenase‐2, which will promote tumor neointima formation and increase tumor cell resistance to PDT. To overcome this issue, Yue et al.^60^ explored the development of an alternating radiation curation‐driven PDT and RNA interference (RNAi) combination therapy that could synergistically inhibit tumor growth and metastasis (Figure 8D). By inducing a high dose of light‐mediated rapid apoptosis and a low dose of light‐mediated efficient VEGF silencing, the combination of PDT and RNAi achieved significant antitumor effects both in vitro and in vivo.

### 6.5 Nanocarriers assisted combination therapy

Although PDT has been rapidly developed to meet the requirements of cancer treatment, the therapeutic efficacy of monotherapy is still limited due to tumor heterogeneity and in vivo environmental complexity. These issues have together accelerated the emergence of multimodal synergistic therapies, offering valuable insight into cancer theranostics. The integration of PDT with varied antitumor strategies, such as chemotherapy, PTT, and immunotherapy, has been reported with satisfactory anticancer performances.

**FIGURE 9:**
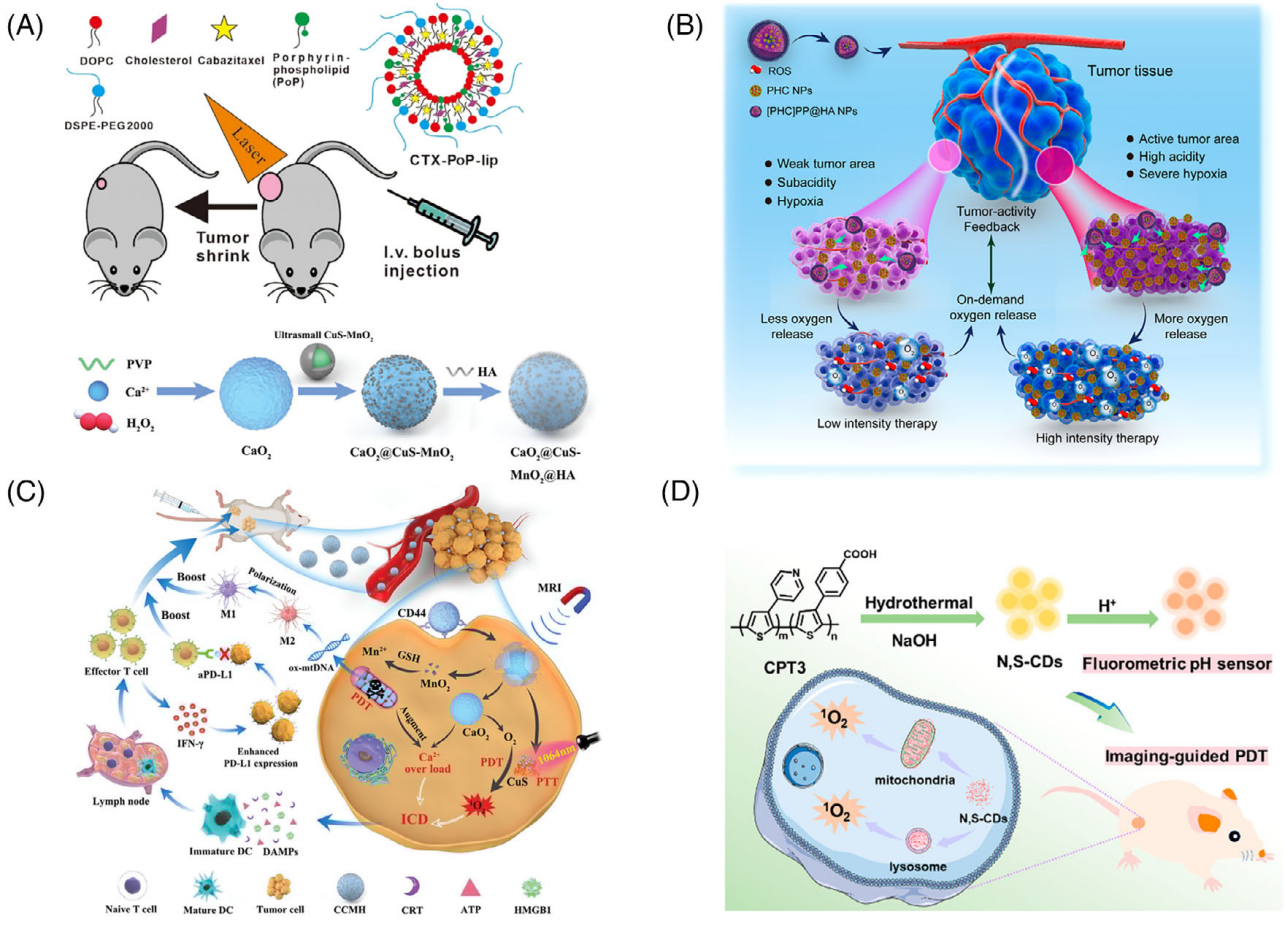
Schematic presentation of nanomaterials assisted combination therapy. (A) Schematic representation of the anticancer chemophototherapy system based on cabazitaxel‐loaded liposomes using porphyrin‐phospholipids. Reproduced with permission.^57^ Copyright 2022, American Chemical Society. (B) Schematic illustration of the design and preparation of small‐sized nanoparticles using carrier polydopamine and photosensitizer Ce6 for synergistic PTT/PDT therapy of tumor. Reproduced with permission.^222^ Copyright 2020, American Chemical Society. (C) Schematic illustration of CaO_2_@CuS–MnO_2_@HA nanoparticles combined with NIR‐II phototherapy for synergistic reinforcing of ICD and transforming tumor‐associated macrophages. Reproduced with permission.^224^ Copyright 2022, Wiley. (D) Illustration of preparing PTP‐based carbon dots and their application for imaging‐guided PDT. Reproduced with permission.^160^ Copyright 2021, American Chemical Society. ATP, adenosine triphosphate; Ca^2+^, calcium; CCMH, CaO_2_@CuS–MnO_2_@HA; CDs, carbon dots; CRT, calreticulin; HMGB1, high mobility group box 1; ICD, immunogenic cell death; MRI, magnetic resonance imaging; PDT, photodynamic therapy; PHC, penetrating and pH‐responsive composite; PoP, porphyrin‐phospholipid; PTT, photothermal therapy; PVP, polyvinylpyrrolidone; ROS, reactive oxygen species.

PDT as a minimally invasive and nontraumatic approach, holds promise for localized tumor eradication. When combined with chemotherapeutic agents, it can achieve enhanced local tumor treatment efficacy. Sun et al.^57^ innovatively developed a strategy to combine the merits of both PDT and chemotherapy. They engineered porphyrin phospholipids and other phospholipids into liposomes, incorporating hydrophobic second‐generation paclitaxel analog cabazitaxel within the phospholipid bilayer (Figure 9A). This unique design enabled the synchronous delivery of the PS and the chemotherapeutic drug, forming a hybrid regimen of chemotherapy and PDT. Upon laser irradiation at a specific wavelength, PDT was triggered alongside the release of paclitaxel analog, yielding a synergistic therapeutic outcome. The obtained system exhibited excellent storage and serum stability, as well as robust drug stability under laser irradiation. This integrated approach demonstrated substantial suppression of tumor growth in the human pancreatic cancer mouse model in comparison with PDT or chemotherapy alone. PDT operates by consuming intracellular O_2_ to produce cytotoxic singlet O_2_, aggravating cellular hypoxia. This characteristic potentially accelerates the discharge of anoxia‐activated prodrugs. Consequently, merging a depletion‐activated chemotherapeutic agent with PDT emerges as an enticing strategy for potent tumor treatment. Wang et al.^213^ designed iRGD‐modified nanoparticles to codeliver the PS ICG and the O_2_‐depleted prodrug tirapazamine, yielding a synergistic therapeutic effect. In a parallel study, Yuan et al.^220^ engineered a photopromoted nanoparticle to release the PS Ce6 and the O_2_‐depleted autophagic prodrug paclitaxel (PTX2‐Azo), resulting in synergistic cancer therapy. These findings underscore how PDT‐driven reactive O_2_ species generation exacerbates cellular hypoxia, consequently enhancing the release of anoxia‐activated prodrugs for superior tumor therapy. In this context, Prof. Zhang's team ingeniously combined these insights and synthesized a novel molecular prodrug, CS‐P. CS‐P interweaves an azaspiracid with a NIR PS through an O_2_‐sensitive azobond. In O_2_‐deprived microenvironments, these azobonds can be reduced, liberating therapeutic agents and PSs. Fluorescence and photoacoustic imaging showcased a gradual surge in tumor‐site fluorescence intensity postinitial dip, illuminating how PDT facilitates drug release. Moreover, CS‐P could effectively target mitochondria, inducing cell death via their destruction. This strategy successfully achieved amplified drug release and synergistic therapeutic efficacy.

Many PSs have both photothermal and photodynamic effects. The cooperation of PDT and PTT has proven to inherit the advantages of low toxicity and side effects of light therapy but also allows them to overcome their respective drawbacks and achieve synergistic effects.^221^ For example, PTT can improve vascular saturation of O_2_ by increasing the rate of concentrated blood flow, and mild thermotherapy can increase membrane permeability and enhance the uptake of PS by tumor cells, thus promoting PDT efficiency and conversely ablating heat‐resistant tumor cells in PTT. Wang et al.^222^ have introduced a pioneering approach that demonstrates remarkable progress in curbing tumor recurrence through a synergistic PTT and PDT strategy. Their study centered around the creation of small‐sized nanoparticles using biocompatible polydopamine as a carrier, adeptly loaded with both hemoglobin to supply O_2_ and the PS Ce6. Subsequently, these small particles were enclosed within acid‐responsive PEG–polyethyleneimine (PEI) micelles and surface‐modified with HA, rendering them a composite PTT/PDT synergistic therapeutic nanocarrier with high permeability and acid‐sensitive release property (Figure 9B). In the tumor's acidic microenvironment, polydopamine was released to engage in PTT, while the released Ce6 could activate O_2_‐mediated PDT, synergistically eradicating tumors under light exposure. Encouragingly, the in vivo antitumor experiments showcased the approach's potency, achieving a tumor inhibition rate exceeding 98% and a mere 8.3% tumor recurrence rate in mice over a 60‐day period. BP nanosheets, a recent class of two‐dimensional nanomaterials discovered after graphene, hold immense potential for diverse applications. As a metal‐free layered semiconductor, BP nanosheets possess a tunable band gap dependent on thickness, enabling absorption across UV and visible spectra, and yielding NIR photothermal properties suitable for PTT. Its folded lattice structure contributes to a higher surface area‐to‐volume ratio, translating to enhanced drug‐loading capacity. BP's distinctive electronic structure also positions it as a proficient PS, capable of generating single‐line O_2_ for PDT. These attributes, along with its unique structure, make BP nanosheets a promising candidate in drug delivery, tumor PTT, and PDT. Chen et al.^223^ introduced an innovative concept of a synergistic photodynamic/photothermal drug delivery system using BP nanosheets. Notably, owing to its multifold structure and negatively charged surfaces, BP nanosheets exhibit a superior drug retention capability, achieving a remarkable 95% loading efficiency of DOX, surpassing previously reported 2D material systems. In vitro and in vivo experiments validated the system's potential as a superior tumor cell eradication strategy, capitalizing on the synergistic fusion of chemotherapy with DOX, photothermal, and photodynamic therapies involving BP nanosheets.

PDT can trigger ICD by initiating endoplasmic reticulum oxidative stress within tumor cells. This arises from the generation of reactive O_2_ species by PSs when exposed to light. Consequently, a substantial amount of calcium reticulum proteins relocates to the cell membrane's surface, accompanied by the release of DAMPs, which includes the extracellular release of heat‐shock proteins (HSPs), HMGB1, and ATP. The exposure of CRT facilitates the binding of antigen‐presenting cells (APCs) to phagocytose dead cells. HSPs act as a family of molecular chaperones sustaining cellular homeostasis. HMGB1 can promote the presentation of tumor‐associated antigens to APCs. ATP acts as a proinflammatory stimulus. Additionally, ICD can further enhance APCs maturation by secreting proinflammatory cytokines. Therefore, ICD can significantly promote the activation of the immune response, which serves as a bridge connecting PDT with immunotherapy. Nevertheless, the efficacy of PDT‐induced immune activation is constrained by factors like the extent of cytotoxic T cell infiltration within tumor tissues and the presence of various immunosuppressive elements. Hence, strategies integrating PDT with immunotherapy are pivotal in advancing the therapeutic impact of PDT. In their recent investigation, Xu and colleagues introduced a dual‐action approach for augmenting ICD via CaO_2_@CuS–MnO_2_@HA nanocomposites (Figure 9C). This innovative strategy addressed the limitations of conventional DAMP inducers in ICD. Grounded in PDT, CuS nanoparticles under NIR irradiation (1064 nm) could engender ^1^O_2_, inducing ICD and simultaneously interfering with mitochondrial calcium buffering. Furthermore, CaO_2_ nanoparticles could undergo hydrolysis within the cells, generating a substantial amount of O_2_ and calcium ions. This dual mechanism could not only amplify PDT efficacy but also trigger a surge of calcium ions during mitochondrial impairment, leading to robust ICD activation. This event instigated the transformation of tumor‐associated macrophages into the M1 subphenotype, reshaping the immunosuppressive TME. The results showed that the nanocomposites activated a potent antitumor immune response in both 4T1 and CT26 tumor models. The integration of PDT with immune checkpoint blockade can potentially amplify the immune response to prevent local tumor recurrence and slow the growth of untreated disease (distal effect).^224^ Previously, Lou et al.^225^ found that repetitive PDT (R‐PDT) using porphyrin lipoprotein as a PS could induce distal effects without the combination of immune checkpoint inhibitors. To understand the mechanism, the immune response induced by therapies such as R‐PDT and the combination of R‐PDT + PD‐1 were investigated in the highly aggressive subcutaneous AE17‐OVA mesothelioma double‐tumor C57BL/6 mouse model. It was found that R‐PDT and R‐PDT + PD‐1 therapies produced a 46‐fold and 61‐fold increase of IL‐6, respectively, suggesting that they could achieve broad innate immune activation. Dendritic cells and macrophages showed increased expression of major histocompatibility complex class II, CD80, and CD86, and there was a greater tendency for antigen presentation in the spleen and distal unirradiated tumor‐draining lymph nodes. At the same time, the proportion of CD^4+^ T cell subsets in the spleen and the frequency of CD^8+^ T cells in distal unirradiated tumor‐draining lymph nodes also increased.

The integrated system for theranostics is a clever integration of precise diagnostic imaging and treatment modalities into one, offering significant advantages over a single diagnostic or treatment modality. Owing to the inherent fluorescence of PS molecules, real‐time tracking of in vivo PS distribution has become feasible. This capability holds immense potential in delineating tumor margins and detecting minuscule tumor clusters, which are imperceptible to the naked eye during surgical procedures. This innovative approach guides meticulous tumor excision, while also facilitating subsequent PDT targeting residual tumor cells, thereby heightening therapeutic efficacy. The amalgamation of fluorescence imaging for intraoperative navigation and PDT has yielded promising clinical outcomes in the realm of cancer treatment. Lu et al.^226^ introduced a novel molecular design strategy for a type of PS, termed A‐D‐A‐D‐A, with an emphasis on manipulating its spatial barrier effect. This manipulation resulted in enhanced emission peaks within the NIR‐II range, crucial for maintaining adequate excitation energy for efficient ^1^O_2_ generation when the PS was in its aggregated state. Through this approach, BNET, a PS of this type, was developed. The ensuing albumin‐conjugated BNET nanoparticles demonstrated commendable traits such as proficient ^1^O_2_ generation, robust photostability, and remarkable biocompatibility. Notably, these BNET nanoparticles exhibited precise NIR‐II imaging specificity in mouse models with in situ colon or pancreatic tumors, and they also displayed notable PDT performance under imaging‐guided conditions. Carbon dots (CDs) have numerous advantages, including facile preparation, customizable optical properties, and excellent biocompatibility. These attributes have driven significant advancements in the realm of CDs for bioimaging and biomedical applications. In the pursuit of effective PDT for cancer treatment, a pivotal objective involves the creation of imaging‐guided, subcellular organelle‐targeted CDs. In this context, Zhao et al.^160^ achieved a significant milestone by synthesizing N, S‐CDs utilizing PTP derivatives as the carbon source (Figure 9D). Remarkably, N, S‐CDs exhibited the capability to infiltrate within tumor cells and accumulate in lysosomes and mitochondria, thereby potentially serving as efficacious PSs for image‐guided PDT. The results showed that N, S‐CDs displayed pH sensitivity, with fluorescence intensity gradually intensifying as the pH drops from 6.25 to 2, and a novel emission peak emerging at around 500 nm. This pH‐responsive fluorescent sensor holds promise for distinguishing between cancer cells and normal cells, thereby augmenting its potential utility.

## 7 CLINICAL STUDIES IN PDT

PDT has been used for cancer treatment for more than 30 years, since its introduction into clinical practice in the early 1900s.^227^ Over the past 30 years, PDT has achieved milestones in the treatment of tumors of the skin,^228^ breast,^229^ lung,^230^ urinary and genitourinary systems (e.g., bladder, urethra, prostate),^231^ and gastrointestinal tract (e.g., esophagus, stomach, rectum).^232^ For example, PSs such as Photofrin^®^ have been successfully marketed for the treatment of esophageal and lung cancers (Table 3). With the help of nanocarriers, it is believed that the clinical application of PDT will be further broadened.

**TABLE 3:** PSs approved or under clinical trials.

Brand name	INN of its active substance	Study name/chemical name	Compound class	Excitation wavelength (nm)	Indication	Current status	References or ClinicalTrials. gov identifier
Photofrin^®^	Porfimer sodium	Hematoporphyrin derivative	Porphyrin	630	Lung, bladder, esophageal, gastric cancer	Approved in over 40 countries	233
Levulan^®^	5‐ALA	5‐Aminolevulinic acid	Precursors of porphyrins	635	Actinic keratoses, basal cell carcinoma, head and neck cancer	Approved in The United States of America and The European Union	234
Metvix^®^	5‐ALA	5‐Aminolevulinic acid	Precursors of porphyrins	635	Actinic keratoses, basal cell carcinoma	Approved in The European Union, Australia, and New Zealand	235
Visudyne^®^	Verteporfin	Benzoporphyrin derivative	Benzoporphyrin	690	Age‐related macular degeneration	Approved in over 70 countries	236
Hematoporph‐yrin Injection^®^	HPD	Hematoporphyrin derivative	Porphyrin	630	Superficial cancers of the oral cavity, bladder, bronchus, lung, digestive system, and precancerous lesions such as leukoplakia, and erythema.	Approved in China	86901037000073
Hemoporfin^®^	HMME	Hematoporphyrin derivatives	Porphyrin	532	Erythroderma	Approved in China	237
Bacteriochlorin^®^	Padoporfin	WST09	Bacteriochlorin	759	Prostate cancer	Approved in The European Union	238
Hexvix^®^	Hexaminolevulinate	Hematoporphyrin derivatives	Precursors of porphyrins	635	Bladder cancer	Approved in over 26 countries	6
Tookad^®^	Padeliporfin	WST‐11	Bacteriochlorin	753	Prostate cancer	Approved in The European Union	http://www.photocure.no
Laserphyrin^®^	Talaporfin sodium	ME2906	Chlorin	664	Early‐stage lung cancer, primary malignant brain tumor, radiotherapy, or local recurrence of persistent esophageal cancer after radiation therapy	Approved in Japan	239
Foscan^®^	Temoporfin	mTHPC	Chlorin	652	Prostate and pancreatic tumors, head and neck cancer	Approved in The European Union	240
SGX301^®^	Synthetic hypericin	SGX301	Hypericin	570–650	Early‐stage cutaneous T‐cell lymphoma	Approved in The United States of America and The European Union	http://www.qltinc.com
							http://www.photocure.no
LUZ11^®^	Redaporfin	LUZ11	Bacteriochlorin	749	Biliary tract cancer	Approved in The European Union	http://www.photocure.no
LUZ11	Redaporfin	LUZ11	Bacteriochlorin	749	Head and neck cancer	Phase 1, Phase 2	NCT02070432^241^
Foscan	Temoporfin	mTHPC	Chlorin	652	Nasopharyngeal carcinoma	Phase 2	NCT01086488^242^
Visudyne	Verteporfin	Benzoporphyrin derivative	Benzoporphyrin	690	Glioblastoma multiforme of brain glioma, sarcomatous	Phase 1	NCT02464761^243^
Pc 4	Silicon phthalocyanine 4	Silicon phthalocyanine 4	Phthalocyanines	675	Recurrent cutaneous T‐cell non‐Hodgkin lymphoma recurrent mycosis fungoides, Stage I cutaneous T‐cell non‐Hodgkin Lymphoma, Stage IA mycosis fungoides, Stage IB mycosis fungoides, Stage II cutaneous T‐cell non‐Hodgkin Lymphoma, Stage IIA mycosis fungoides	Phase 1	NCT01800838^244^
HPPH	Photochlor	HPPH	Chlorin	665	Head and neck cancer	Phase 1	NCT00675233^245^
SGX301^®^	Synthetic hypericin	SGX301	Hypericin	532	Cutaneous T‐cell lymphoma	Phase 3	NCT02448381^246^
LS 11	Talaporfin sodium	ME2906	Chlorin	664	Liver metastasis, pelvic cancer, head and neck cancer, sarcoma, rectal cancer, breast cancer, colorectal cancer, oral cancer	Phase 1, Phase 2	NCT02070432^247^

Abbreviations: HMME, hematoporphyrin mono‐methylether; HPPH, 2‐[1‐hexyloxyethyl]−2‐devinyl pyropheophorbide‐a; INN, the International Non‐Proprietary Name; mTHPC, 5,10,15,20‐tetrakis(m‐hydroxyphenyl)chlorin.

PDT can be combined with radiotherapy,^255^ chemotherapy,^256^ and surgery^231^ because of its advantages, such as a noninvasive treatment process and unique treatment mechanism (Table 4). It is worth mentioning that the combination of radiotherapy and PDT seems to be an attractive therapy because PDT can be used as an effective remedial treatment after radiotherapy. Yano et al.^257^ used talaporfin sodium‐based PDT to treat patients with histologically proven local failure after chemoradiotherapy or radiotherapy. The study showed that PDT treatment was excellent. Salvage PDT with talaporfin sodium showed a high local complete response rate, and no significant skin phototoxicity or PDT‐related grade 3 or worse nonhematologic toxicity was observed. In another study, Liu et al.^258^ randomly assigned 40 patients with recurrent breast cancer to receive radiotherapy alone or PDT‐radiotherapy combination therapy. There was no statistical difference in the response rates between the two groups; however, more patients had a complete response in the PDT + radiotherapy combination group (50%) than in the radiotherapy alone group (20%), and the median time to complete response was reduced from 175.2 days in the PDT + radiotherapy combination group to 109.6 days in the radiotherapy group (*p* = 0.001). Apparently, the addition of PDT to radiotherapy has been shown to be beneficial in improving antitumor effects, and this combination therapy may be applied to reduce the time required to receive radiotherapy.

**TABLE 4:** Some clinically available photodynamic combination therapies.

Application	Current status	Excitation wavelength (nm)	Combination therapy	ClinicalTrials. gov identifier
Nonmuscle invasive bladder cancer refractory to Bacillus Calmette‐Guérin vaccine (BCG)	Phase 2	520	Chemotherapy (TLD‐1433 Bladder infusion) + PDT	NCT03945162^247^
Mesotheliomas pleural, malignant pleural, mesothelioma	Phase 2	400–500	Immunotherapy + Pleural 5‐ALA‐based PDT	NCT04400539^248^
Microinvasive squamous cell carcinoma	Phase 1	632	Ablative fractional laser (AFL) + PDT	NCT02666534^249^
Choroidal melanoma	NA	810	transpupillary thermotherapy + ICG‐based PDT	NCT01253759^250^
Locally advanced lung carcinoma, non‐small cell lung carcinoma, small cell lung carcinoma, Stage III lung cancer American Joint Committee on Cancer (AJCC) v8, Stage IIIA lung cancer AJCC v8, Stage IIIB lung cancer AJCC v8, Stage IIIC lung cancer AJCC v8	Phase 1	630	Ultrasound + Operative treatment (endobronchial ultrasound with transbronchial needle) + PDT	NCT03735095^251^
	Phase 2			
Nodular basal cell carcinoma	Phase 1	632	Er:YAG AFL + PDT	NCT02018679^252^
Brain and central nervous system tumor	Phase 3	630	Surgical procedure, chemoradiotherapy, chemotherapy with or without PDT	NCT00003788^253^
Epithelioid malignant pleural mesothelioma	Phase 2	630	RP (radical pleurectomy) + Photofrin‐based PDT	NCT02153229^254^

Abbreviations: 5‐ALA, 5‐aminolevulinic acid; Er, YAG AFL: a laser model; ICG, indocyanine green; NA, not reported in the literature; PDT, photodynamic therapy; TLD, a novel ruthenium‐based PS.

PDT is also an effective adjunct to enhanced chemotherapy or surgical therapy. Ryu et al.^259^ found that patients with advanced malignant pleural mesothelioma survived for 27 months after combined chemotherapy and PDT treatment; their symptoms improved, and their survival time was extended. In another study, Filonenko et al.^231^ selected 45 eligible subjects treated for nonmuscle invasive bladder cancer at 3 clinical centers according to clinical protocol No. 10/1‐(FDT‐ALA)−2007, where patients underwent cystourethrectomy with PDT as an antirecurrence treatment. The study found that patients after the treatment had a reduced recurrence rate of superficial bladder cancer (22%) at the 1‐year follow‐up.

Despite the potential of PDT in clinical applications, its current utilization is primarily limited to superficial tissue diseases due to the constraints of classic PSs.^260^, ^261^ With the rapid advancement of nanocarriers, this technology demonstrates its potential in overcoming the limitations of conventional PSs and even expanding the applications of PDT.^262^, ^263^

## 8 CONCLUSIONS AND FUTURE PERSPECTIVES

The heterogeneity of tumor tissues is severely hampering human efforts to conquer cancer. As a temporally and spatially precisely controllable, noninvasive, and potentially highly efficient method of phototherapy, PDT has developed a reputation as one of the favorable therapeutic strategies at the forefront of oncology. The three components of PDT primarily include PSs, O_2_, and light, in which PSs play a master role in the therapeutic effects of PDT, and the breakthrough of the bottleneck in the application of PDT also largely depends on the research progress of PSs. Consequently, researchers in the area of PDT have focused on the exploitation of PSs. Currently, the PSs being studied and employed are largely categorized into three generations, and particularly the third generation of PSs integrated with nanocarriers has attained satisfactory achievements, which breaks through the application limitations of the first and second generations. Furthermore, PSs can also be divided into two categories, *viz*. inorganic compounds and organic compounds, which are activated in different ways to perform therapeutic effects. The cooperation of nanocarriers and PDT in tumor management not only allows PDT light source to penetrate deep tumors, altering the limitation of PDT application in clinics which is mostly applying to superficial tumors, but also precisely targeting the tumor lesion area through passive targeting, active targeting, and so on, simultaneously alleviating the hypoxia of TME and reversing the hypoxia‐resistance of conventional PDT in tumor management. Therefore, PDT in collaboration with nanocarriers has tremendous application prospects in overcoming current challenges.

Although the nanocarriers‐based PDT has addressed the problem of poor therapeutical effects of traditional PDT, the safety of PDT remains unaddressed, which is also a primary challenge for the future of PDT. The safety issues including eliciting pain or burning sensations,^264^ allergic reactions,^265^ and genotoxicity ascribed to DNA oxidative damage and breaks,^266^ which must be addressed. The drug resistance mechanism of PDT is still undefined.^267^ It is not enough for the nanocarriers‐based PDT to enhance the therapeutic effects of PDT merely through performing the PS modification and increasing the penetration of the light source into the deep tumors. In the future, the application of nanocarriers‐based PDT should be utilized to profoundly elaborate the resistance mechanism of tumor cells from multiple perspectives, fundamentally tackling the phenomenon of poor therapeutic effects of PDT.

There are still other several challenges associated with the clinical application of nanocarriers‐based PDT as described below: (1) The long‐term or excessive administration of nanocarriers may potentially lead to cumulative toxicity in humans; therefore, it is imperative to thoroughly and comprehensively evaluate the safety and biocompatibility of nanocarriers before their clinical implementation. (2) The release behavior and pharmacokinetics of nano‐PSs necessitate comprehensive investigation through the standardization of PDT regimens in clinical settings and the development of specialized devices.^268^ (3) The efficacy and mechanisms underlying different combination therapies involving nanocarriers‐based PDT remain unclear and require further elucidation. (4) Further research and validation are needed regarding large‐scale production feasibility for nano‐PSs.^269^ (5) Although third generation PSs in collaboration with nanocarriers have broadened the scope of clinical application of PDT, research on third generation PSs is still mainly in the laboratory phase. There are considerable differences in the physiological and pathological features between experimental animals and patients. It is challenging to judge whether the therapeutic effects can be extrapolated into patients based on the obtained data from experimental animals; hence, the clinical translation will still require more efforts.^270^

Nevertheless, successful clinical studies on classical PSs are expected to inspire researchers to overcome challenges associated with nanocarriers‐based PDTs for cancer therapy. We look forward to the development of PDT theory and practice in the field of tumor treatment to achieve optimal biosafety and therapeutic effects and create new opportunities for the survival of cancer patients.

## AUTHOR CONTRIBUTIONS

Paper writing, artworks preparation, and literature survey: Wanchen Zhao, Liqing Wang, and Meihong Zhang. *Capturing graphics copyrights*: Zhiqi Liu. *Conceptualization, proof‐reading, and fund‐seeking*: Guilan Quan, Chao Lu, and Zhengwei Huang. *Manuscript polishing and program management*: Chuanbin Wu and Xin Pan. All authors have read and approved the final manuscript.

## CONFLICT OF INTEREST STATEMENT

There are no conflict of interest to declare.

## ETHICS STATEMENT

Not applicable.

## Data Availability

Not applicable.
